# Topology Analysis of Social Networks Extracted from Literature

**DOI:** 10.1371/journal.pone.0126470

**Published:** 2015-06-03

**Authors:** Michaël C. Waumans, Thibaut Nicodème, Hugues Bersini

**Affiliations:** École polytechnique de Bruxelles CoDE-IRIDIA, ULB, Brussels, Belgium; Universidad Rey Juan Carlos, SPAIN

## Abstract

In a world where complex networks are an increasingly important part of science, it is interesting to question how the new reading of social realities they provide applies to our cultural background and in particular, popular culture. Are authors of successful novels able to reproduce social networks faithful to the ones found in reality? Is there any common trend connecting an author’s oeuvre, or a genre of fiction? Such an analysis could provide new insight on how we, as a culture, perceive human interactions and consume media. The purpose of the work presented in this paper is to define the signature of a novel’s story based on the topological analysis of its social network of characters. For this purpose, an automated tool was built that analyses the dialogs in novels, identifies characters and computes their relationships in a time-dependent manner in order to assess the network’s evolution over the course of the story.

## Introduction

The recipe of the Harry Potter saga’s [1–7] success might reside in part in the very unique way its author has installed a familiar kind of social network in a fantasy world. In order for the reader to be seduced by the story of any novel, the social network narrated in the book must not be too distinct from the ones typically found in real life. Interestingly enough, a novel respectful of the social reality of its time also constitutes an interesting temporal compression (many years of the life of many characters held in but one, or a few, books) that allows clever text mining and natural language algorithms to more easily catch the main features of the social network depicted in the novel: its topology (i.e. its degree distribution: is Harry Potter social network scale-free?), its clustering degree (are the friends of Harry friends themselves?) [48] and the way it is being constructed in time (does the social network grow in a random way or does it follow some form of preferential attachment? [49]). Not only is it possible at the end of a book (or the series) to extract and analyse the complete topology of the characters relationship, but it is as well possible to follow the way new characters enter this social network and connect to the existing nodes. Accordingly, a first investigation described in this paper is the study of the various social networks (both their static features and their dynamic nature) found in several popular series and one classic: The seven “Harry Potter” books by J.K. Rowling, the (presently) five “A Song of Ice and Fire” [8–12] books by George R. R. Martin, the three “His Dark Materials” [13–15] books by Philip Pullman, the (presently) three “Lunar Chronicles” books by Marissa Meyer [16–18], the six “The Mortal Instruments” books by Cassandra Clare [19–24], the three “Liveship Traders” [25–27] and four “Rain Wild Chronicles” [28–31] books by Robin Hobb, the fifteen “The Wheel of Time” books by Robert Jordan [32–46], and “Les Misérables” [47] by Victor Hugo.

Our approach starts with an automated construction of the social networks, based on the processing of dialogs in the text. The characters intervening in each conversations are identified, and a network is formed between them based on these interactions. For instance, if in a given part of the text Harry speaks to Hermione, a new link will connect Harry to Hermione in the evolving social network if there were none. Similarly, the first time Jean Valjean addresses Cosette, both the new node “Cosette” and its new link to “Valjean” will be added. The temporal evolution of the network is assumed to follow the succession of dialogs in the books, meaning that new characters appear in a network as soon as they are involved in a conversation. However, we do not believe such an assumption has a major impact on the presented results.

The various steps leading to the creation of this network, as well as the finished social network, provide with a series of key features that form what appears to be a signature of each story, characterizing important elements relating to it, such as the scope, the number of protagonists, and even the author’s style and reading level (for instance, whether or not an author assumes that the reader can keep track of the speakers in a conversation, or the relative proportion of narration within a conversation). This series of attributes leads to a way to characterize each book, and even draw parallels between several of them.

The rest of the article first summarizes the main assumptions and key decisions taken to automatically construct and follow in time the social networks out of the several books. Then, a more complete and sufficiently detailed technological description of the algorithmic steps will be presented in order for any interested reader to easily perform a very similar analysis of their favourite books or series. Finally, the results will be presented and discussed for the forty-seven best-selling books in terms of typical network measures (size, clustering and degree distribution) and the way these networks evolve in time (preferential attachment).

## The main assumptions and algorithmic ideas

First of all, the software technologies presented in this paper should be as automated and user-friendly as possible for anyone interested to easily extract the final social network and its time evolution out of their favourite book. All the software tools and the way to use them will be made available in the bibliography of the paper. The full extraction starts with a text version of the complete book or the complete series. We have arbitrarily restricted our analysis to forty-seven books, but nothing prevents to easily move ahead and enlarge our statistic samples to any size.

The key assumption to construct social networks lies in the exploitation of dialogs and successions of dialogs to automate the extraction in time of the nodes and the links. It is far from surprising to base a social network analysis upon communication among the nodes, since the most common interactive mode between people remains verbal communication. Dialogs are also generally easy to detect and to separate; however, indirect speech will be ignored as a result, as it is syntactically impossible to distinguish from regular narration.

An important algorithmic step of the full protocol is to first isolate one single dialog and insert it in a larger piece of text (including some surrounding narration) referred to as its context. The following key step is to identify the speaker in this context. Then this speaker is connected to the characters he speaks to, and possibly to some others not directly present but mentioned during this dialog. The connectivity pattern to associate with this dialog will be described next but could be: the speakers with all the others, or a fully connected sub-graph (all characters with all).

**Fig 1:**
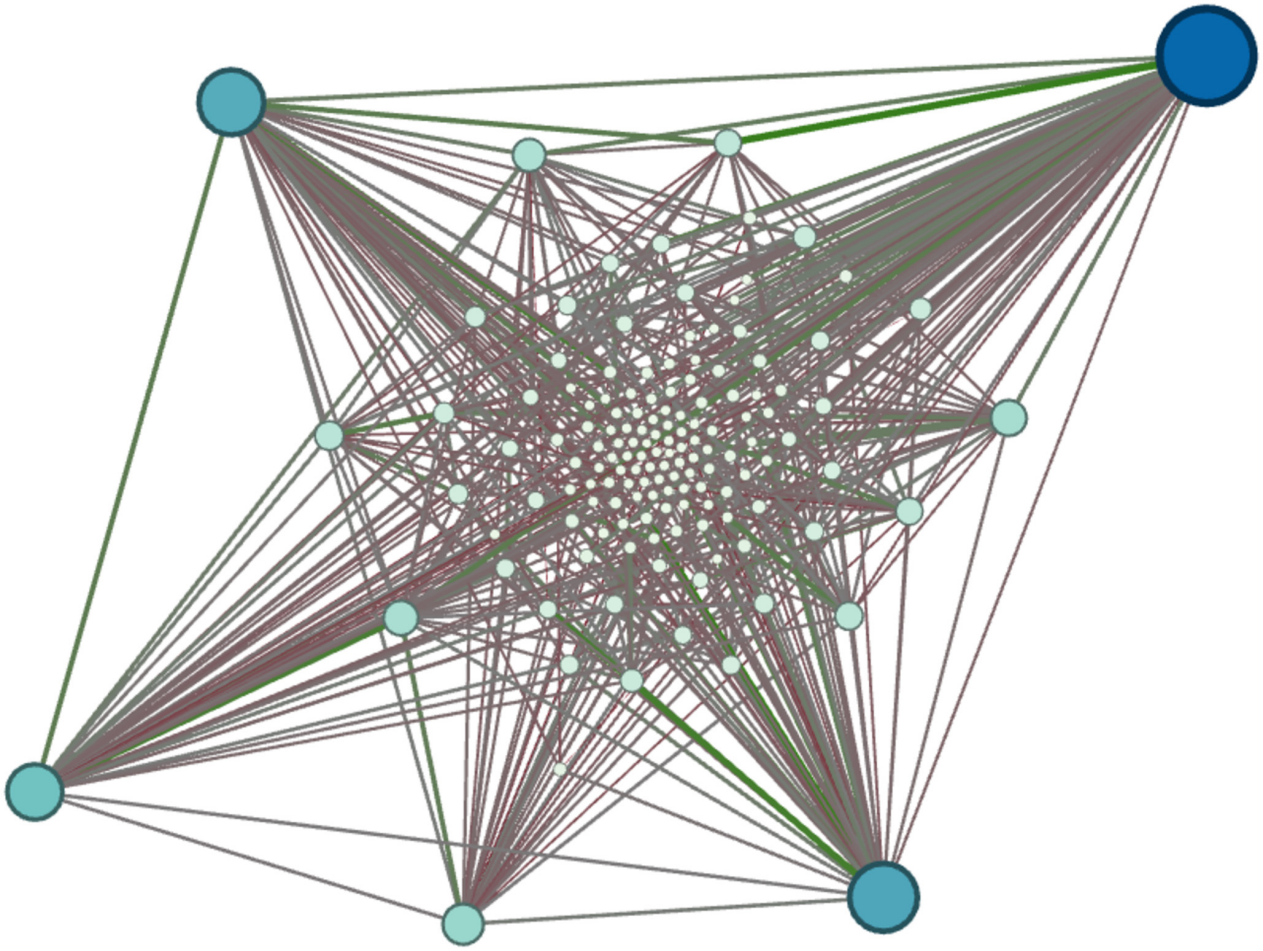
Network Extracted from the book “Harry Potter and the Philosopher’s Stone” [1] with sentiment scores (Produced with NetworkX [50]).

Incorporated in the algorithm is the possibility to weight or colour the links (as can be seen in the network Fig 1) as a result of a sentimental analysis performed on the basis of the words found in the context. In this paper, this additional description will be of no use as our focus is primarily on topology, while for a further characterization of the author’s style or a study extended to weighted graphs, this additional data could be very profitable.

One last assumption is to follow the network construction in time as a succession of the contexts just described above. This is a simplification: the events in a novel are not always told in chronological order. A classical figure of style is the use of flash-backs such as, for instance, at the heart of the narration in “les Misérables”; similarly, temporal inconsistencies may occur with shifting points of view, as are employed in “A Song of Ice and Fire”. Nonetheless, our interest lies mainly in at what point in the story each new character is introduced, and who they are originally attached to when introduced, to verify that a preferential attachment occurs; with this objective and restrictive frame of analysis (the social network’s evolution is only considered over the course of the book’s story, ignoring all backstory and previously-established continuity), this first-order approximation is expected to hold.

## Implementation

The principle of social network extraction from literature may seem simple in theory but is a very complex task in practice that involves many disciplines such as Natural Language Processing, Named-entity recognition, Co-references resolution, Aliases association, etc. Different steps are required to tackle the task properly, they are reviewed in detail in the following sections but an introduction of the important notions is first required.

An author tells a story by switching between descriptions of the events occurring during the story (i.e. the narration) and descriptions of the conversations happening between the different ***characters*** involved. Both are important as they provide information about the characters intervening in the action, but the identification and analysis of the conversations provide a more precise description of the way social links are built through the storytelling.

**Fig 2:**
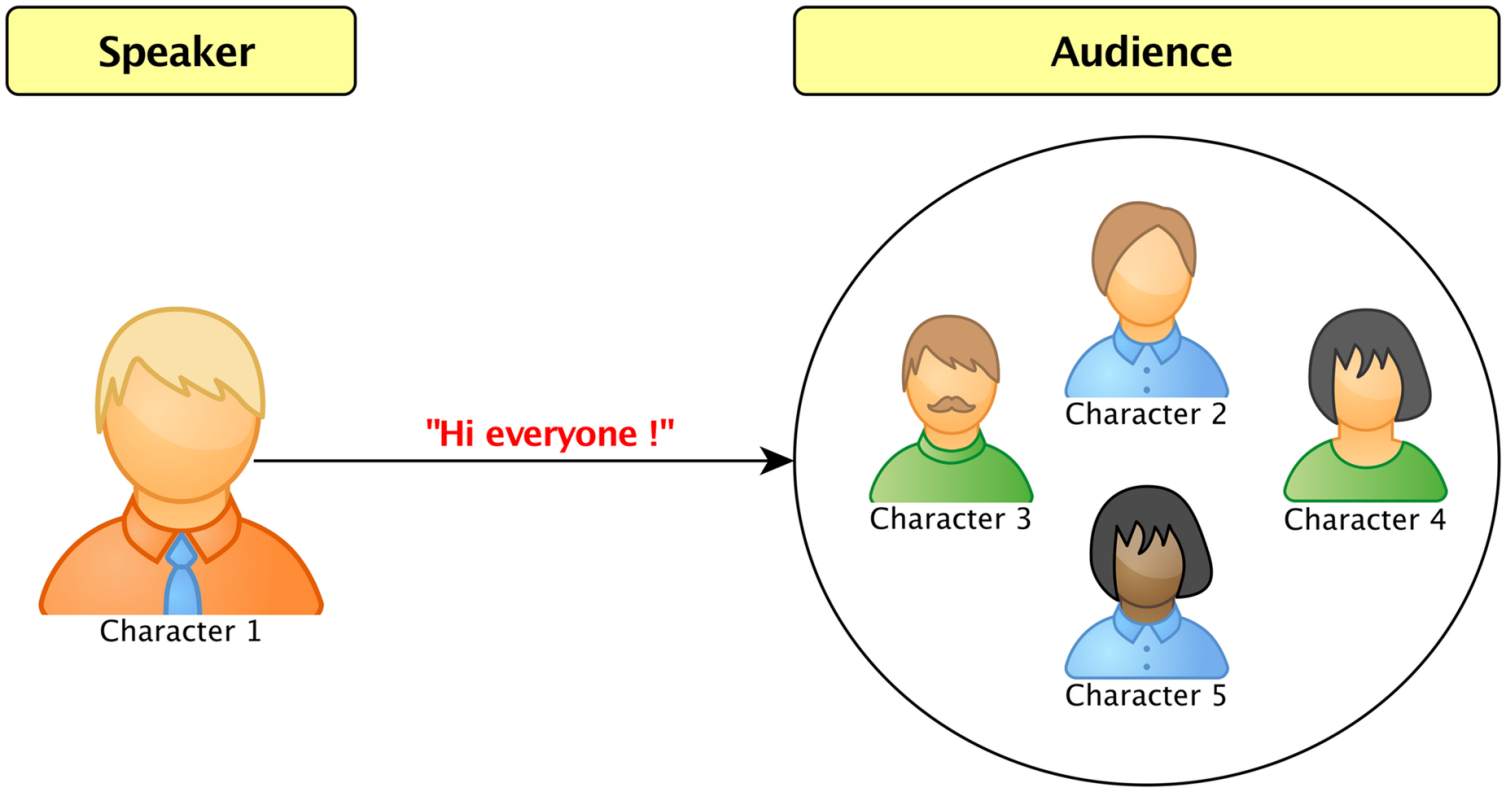
Schematic representation of one interaction between characters.

**Fig 3:**
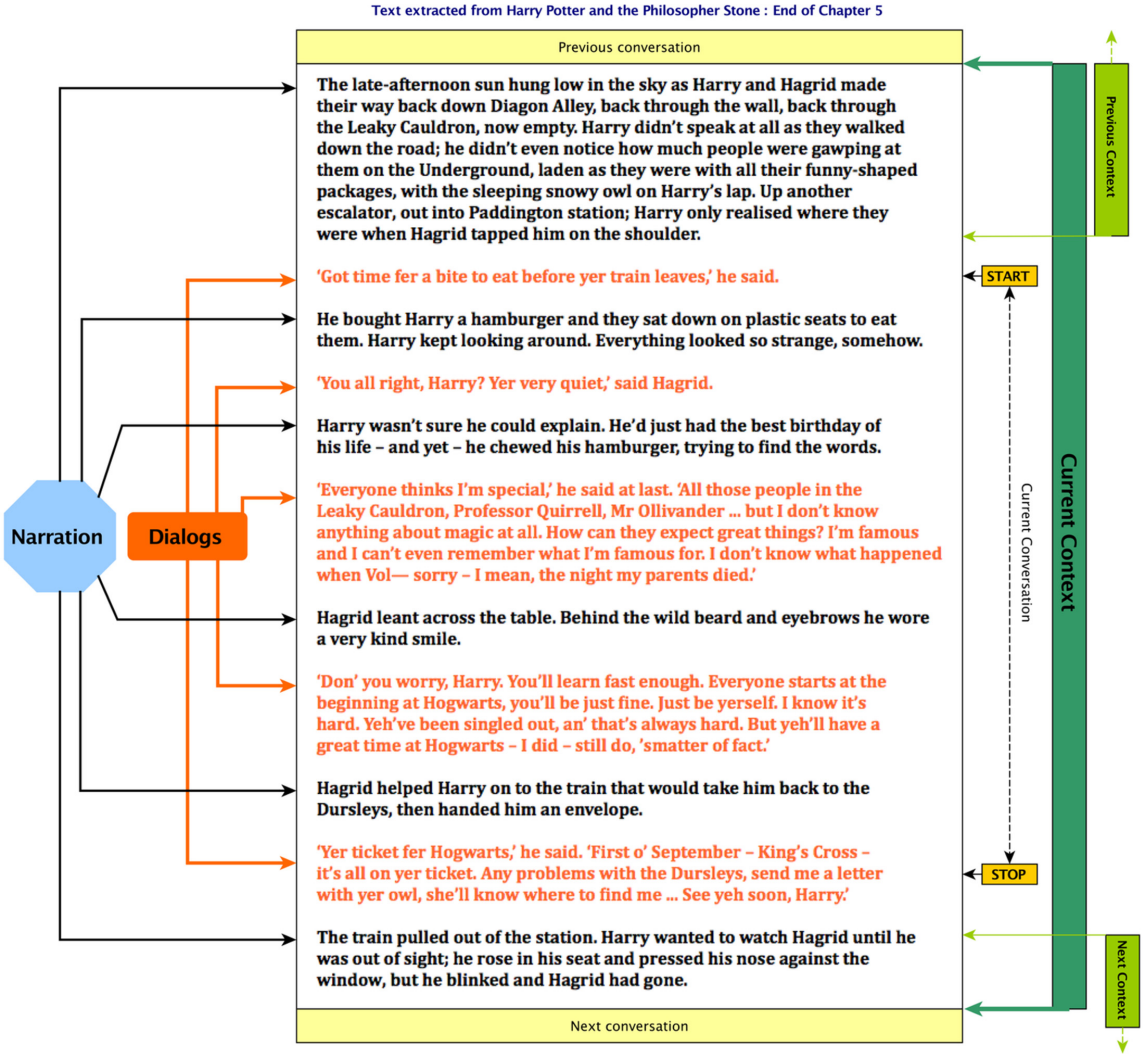
Text extract of a conversation from “Harry Potter and The Philosopher’s Stone” [1] displaying the different elements manipulated (i.e. Narration, dialogs, context, conversation,…) and their relation with each other.

Each ***conversation*** depicted by the author consists in a succession of ***dialogs*** usually indicated in the text by double quotes (See Figs 2 and 3 for examples). As in real life, when someone speaks (the ***speaker***), he addresses himself to one or more persons; ***the audience*** (See Fig 2).

One last notion must be introduced, the ***context*** in which a conversation takes place. As explained before, the author switches between conversation and narration all trough the storytelling and even during a conversation to provide the reader with more information about the context in which it takes place (i.e. the location, who is involved, who they are… See Fig 3 for a practical example). The analysis of the context is necessary to differentiate between the characters participating in a conversation from those who are just mentioned in it (e.g. “I saw Ronald this morning”: Ronald is not actually participating to the conversation but is mentioned nonetheless), and to identify the speakers even though their actual last name, first name or alias is not being used.

The speaker is usually identifiable (subject identification inside a dialog by means of grammatical analysis. See section) as well as the characters to whom he talks by analysing the entire conversion with all the dialogs it contains. The challenge resides in the identification of all the names that refer to each character, and building a network dynamically using the information extracted from the conversations.

To summarize, a novel is composed of many conversations that describe the social interactions between all the characters involved in the action at the specific point in time. Building a network by extracting those information is a complex succession of operations necessary to identify those characters and the way they relate to each other (i.e. who talks to whom).

**Lexicon**:

- ***Character:*** Person that participates to the story.
- ***Speaker:*** Character that speaks to other characters involved.
- ***Audience:*** Characters that listen to a speaker.
- ***Dialog:*** Line of text occurring in a novel denoted by double quotes to indicate that someone is speaking.
- ***Conversation:*** Succession of dialogs representing a conversation between two or more characters.
- ***Context:*** Before, during as well as after a conversation, the author may tell us more about the context in which a conversation takes place. This is what we call the context.

**Fig 4:**
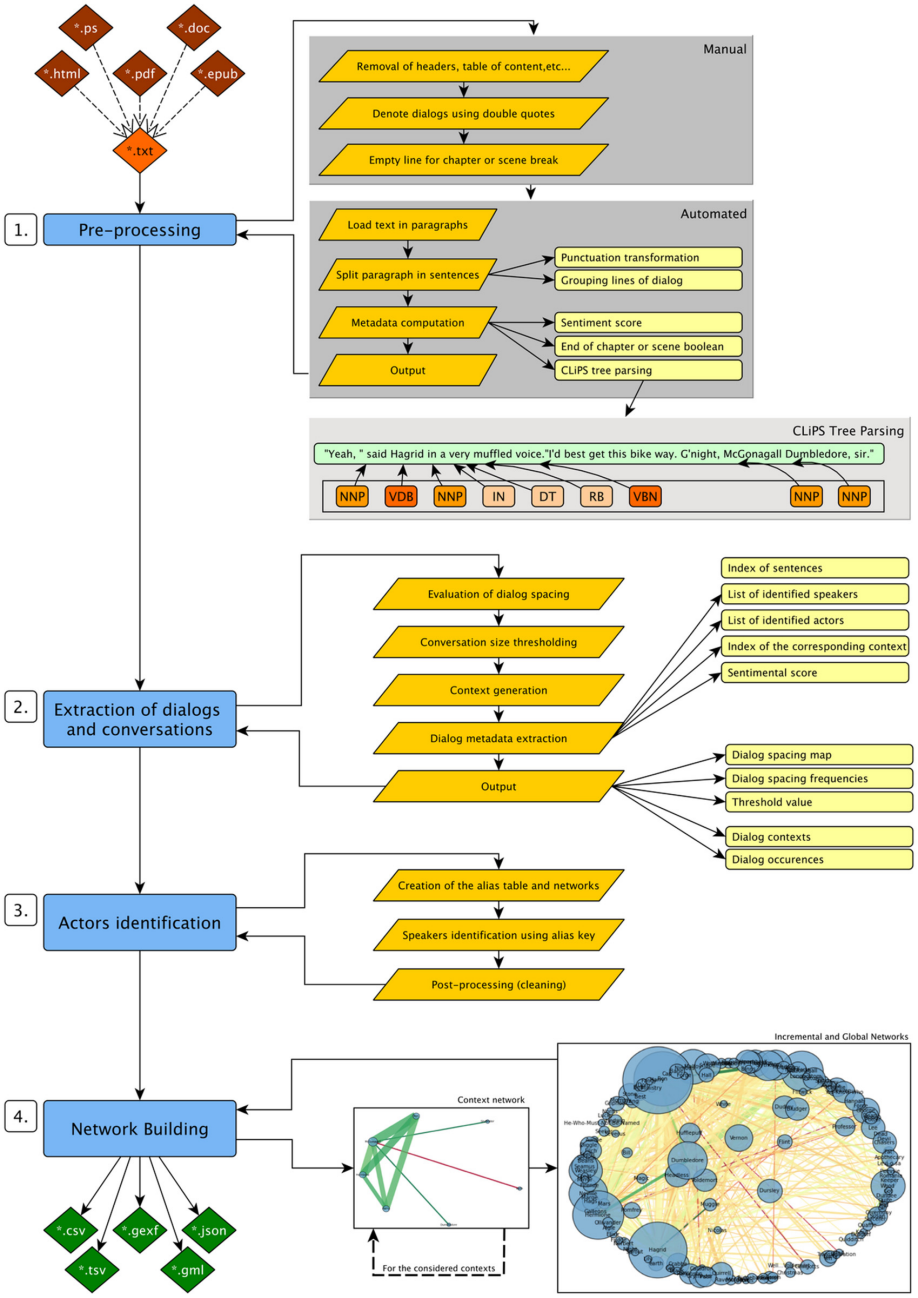
Schematic view of the entire algorithm’s process.

To reach this goal an algorithm composed of four consecutive steps was developed: Pre-processing, Extraction of dialogs and conversations, Characters identification and Network building. They are composed of multiple sub operations as depicted in Fig 4 that are detailed in the following sections.

### Pre-processing

The text to analyse has to respect a specific format to be processable by the algorithm, formatting it properly is the goal of this step. The pre-processing involves manual intervention on the raw text for some aspects (i.e. Removal of headers, table of content,…) and the application of automated procedures to extract the necessary information from the novel.

**Manual pre-processing** The manual step consists in the acquisition of a proper raw text file. The actual text to analyse may be found in different file formats (i.e. HTML, DOC, EPUB, PS, PDF,…) that need to be converted to TXT. (The software Calibre [51] was used for this purpose). The books may also be formatted in different ways (i.e. Use of double quotes or simple quotes to indicate dialogs, use of multiple spacing or specific formatting to indicate the chapters,…) that are not appropriate for an automated processing to take place. At the end of this manual step, whatever the way chosen to apply those modifications, the text must answer only two requirements:

- *Use double quotes to denote dialogs (single quotes for apostrophes)*.
- *Indicate chapter or scene breaks using empty lines with all other empty lines removed*.

**Automated pre-processing** The next step is fully automated. It takes the cleaned raw TXT file as input to compute various information detailed here:

1. Split the text in sentences using the CLiPS Pattern [52] tree parser with two modifications applied to the content. First, some punctuation marks are transformed into others that are recognizable by the tree parser function. Then, the multiple lines forming one dialog are grouped in a same entity (by forcing an even number of quotation marks in a single entity).
2. A sentiment score is computed for each line of text using the built-in opinion mining tool of CLiPS. (Other libraries like NLTK [52, 53, 60] with SentiWordNet [54–57] or CoreNLP [58] were considered, but CLiPS was retained as final solution for this implementation as it suited best the needs of this algorithm). Also, if a scene break or chapter break is found, it is indicated by a boolean value.
3. Produce a parse tree using the CLiPS Tree parser for each sentence and do a division into chunks (i.e: Chunking consists in the division of a text in parts of word that are syntactically correlated. For examples, see Fig 3 in the section ‘CLiPS Tree Parsing’ and the section ‘Characters identification and alias resolution’). This will also allow the determination of the grammatical roles of the words composing the sentences. (i.e. Subject, verb, complement,…).
4. Save the metadata computed on files for later use.

To sum up, this part of the algorithm is concerned with the cleaning of the text as well as the preparation of numerous metadata used for the next steps of the program. Those metadata do include, for each line of text, the sentiment score, the parse tree, the identification of scene or chapter break and the index. (i.e. Each line of text is identified by its index, the number of the line inside the entire novel)

### Extraction of dialogs and conversations

**Fig 5:**
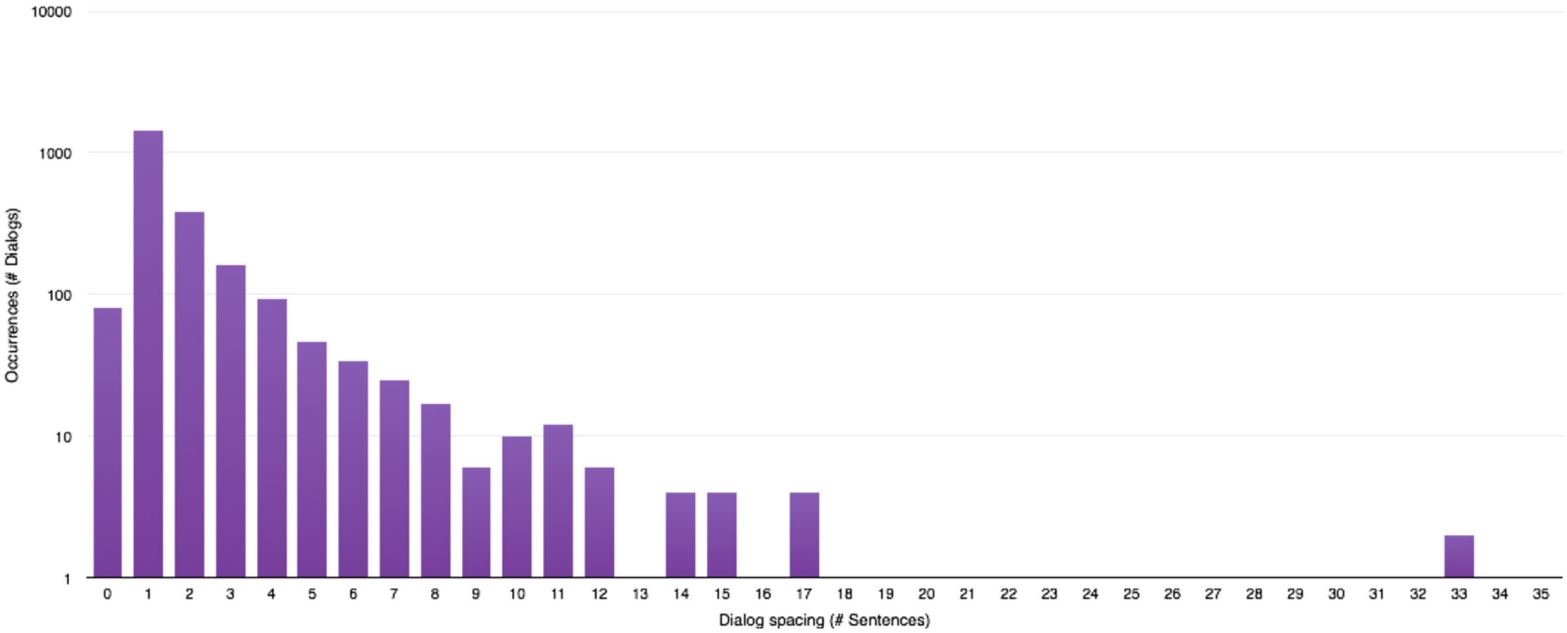
Dialog spacing distribution in “Harry Potter and the Philosopher’s Stone” [1].

**Fig 6:**
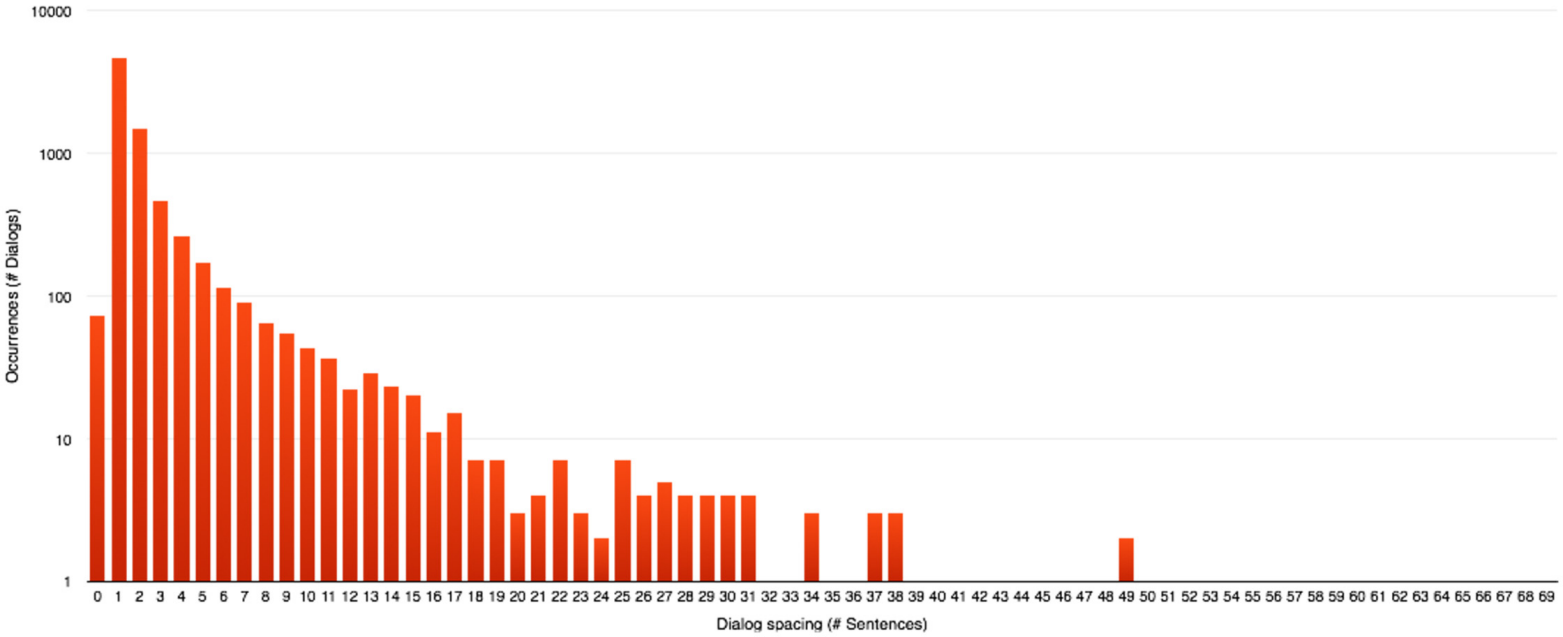
Dialog spacing distribution in “A Game of Thrones” [8].

**Fig 7:**
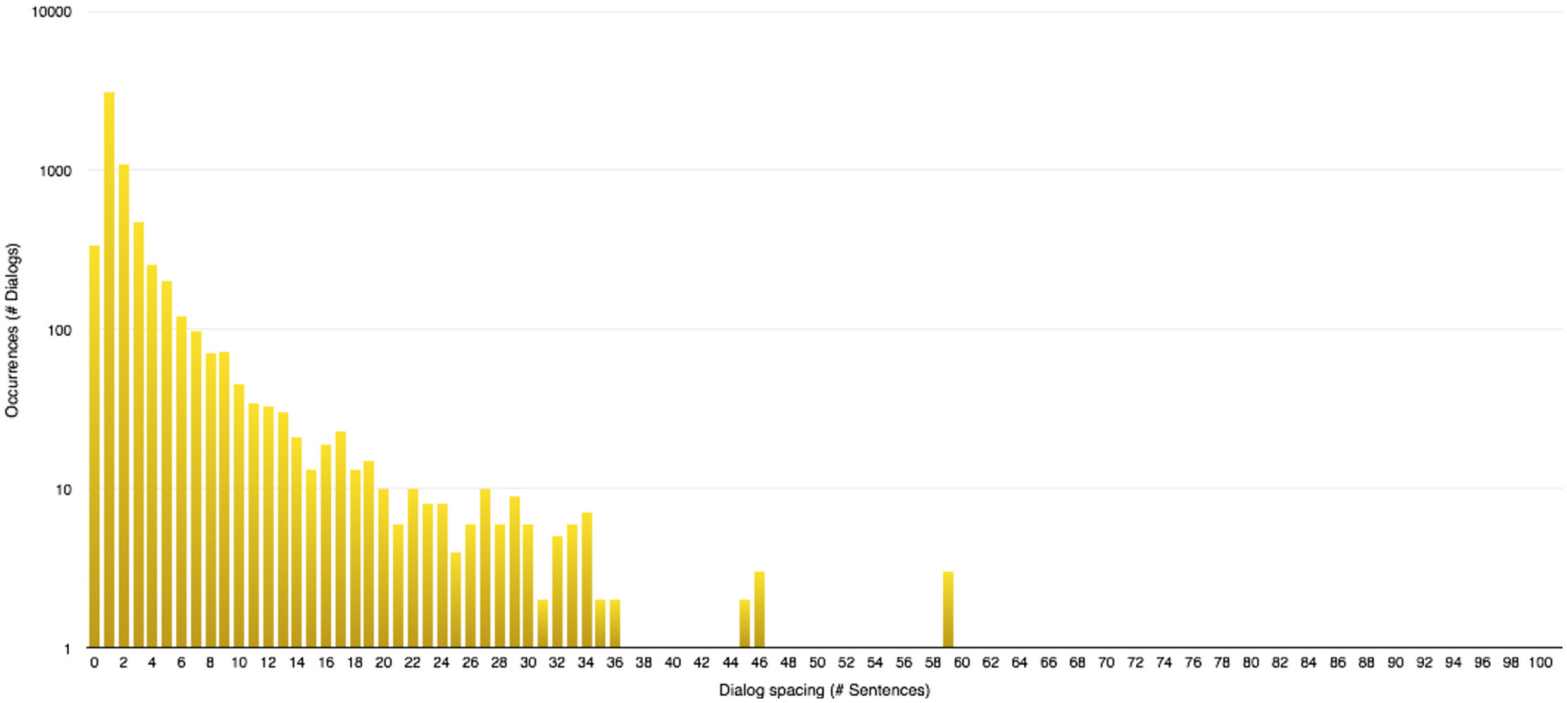
Dialog spacing distribution in “Les Misérables” [47].

**Fig 8:**
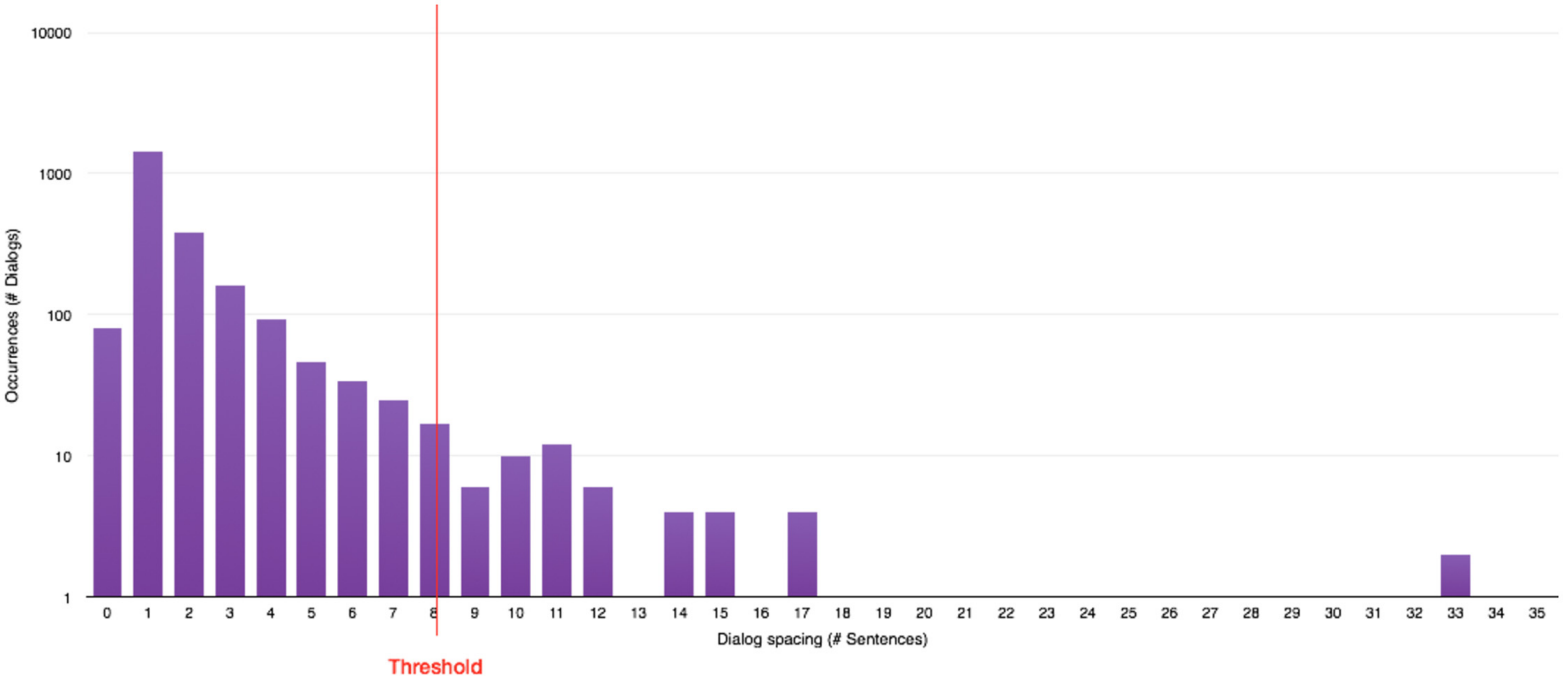
Dialog spacing distribution in “Harry Potter and the Philosopher’s Stone” [1] with the computed threshold.

**Fig 9:**
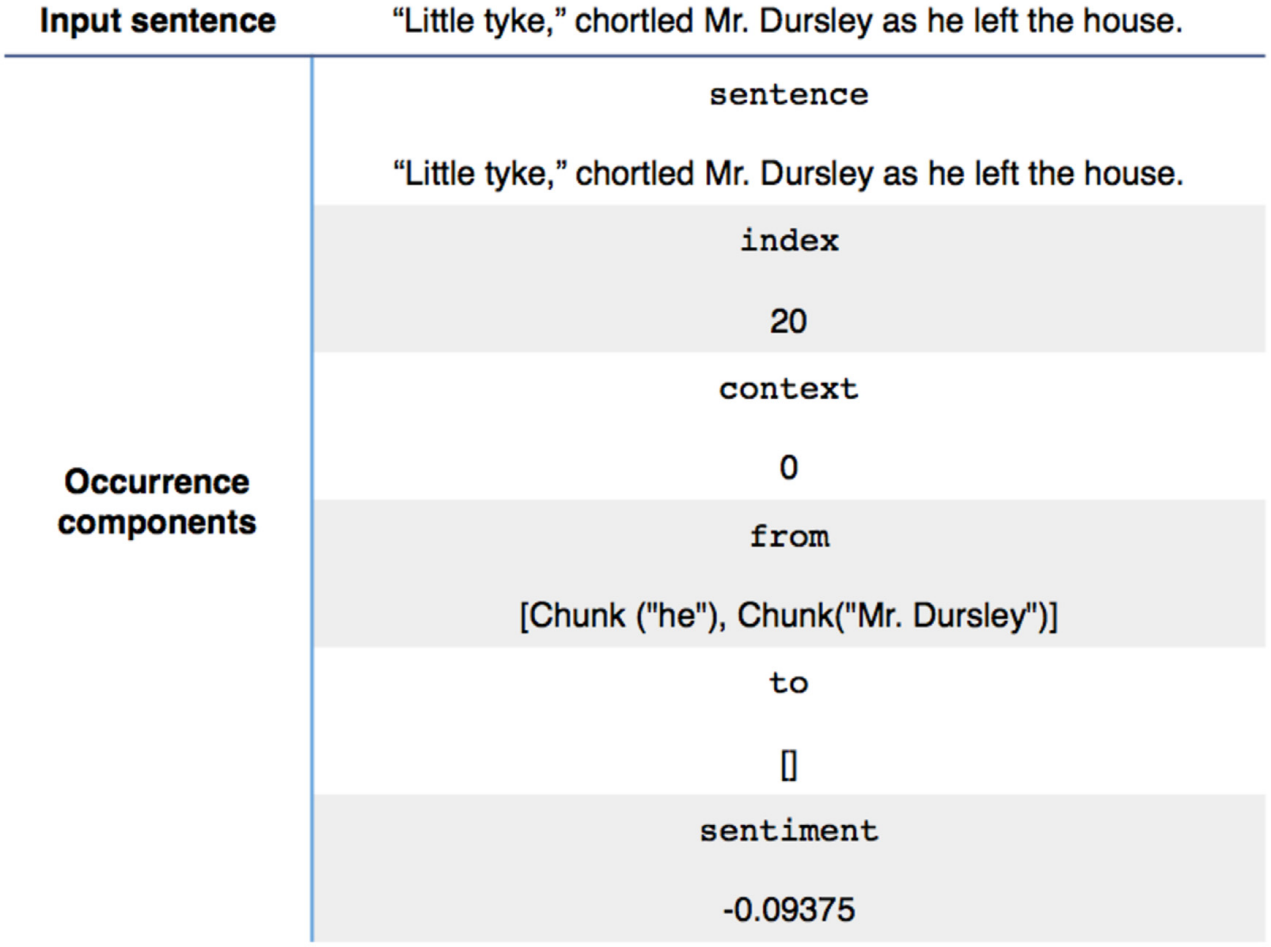
Example of input sentence and it’s related metadata.

A novel tells us a story in which characters interact at different moments in time. While reading a book, we are able to differentiate the contexts that take place easily, as well as the different dialogs and members of the audience. This is much harder for a program to tackle without a perfect knowledge of the language used. This step tackles the task of solving this aspect of the problematic, it identifies the dialogs and conversations in each dialog and also extracts the context of a conversation. The algorithm proceeds as follows:

1. **Evaluating dialog spacing:** This consists in a simple counting operation of the number of sentences present between two lines of dialog.
  To allow a proper division of the story into conversations using an automated process, the values of dialog spacing are analysed during the next step to compute a threshold indicating when one conversation must start and end. Drawing the distribution of those values yields histograms presented in Figs 5, 6 and 7.
  A few general conclusions can be derived from these results. First, the overall shape of the distribution (zeroes excluded since they represent chapter or scene breaks) remains the same and shows a rapidly decreasing profile. Second, each author appears to have a different typical profile as it was observed during the analysis of other series like “Game of Thrones” ([8–12]). This dialog spacing is a distinctive mark of the author’s style. Indeed some authors are more prone to write long sequences of narration, whereas others tend to do the opposite, showing respectively higher or lower value of dialog spacing. Third, the author is more important to the profile than the length of the novel is, even though we reach higher values of spacing in longer novels regardless of the author.
2. **Conversation size thresholding:** Using the values of dialog spacing, a threshold is computed to evaluate the maximum distance between two lines of dialog belonging to the same conversation.
  Unsurprisingly, the frequency distribution heavily favours small numbers since lines of dialog tend to be clustered in conversations. Such observation indicates that it is possible to extract most conversations automatically by relying on a threshold value of dialog spacing. This threshold corresponds to the usual number of spacing (i.e. Sentences of story telling) found between two dialogs. After the analysis of a few books, an empirical formula was derived. Namely, the threshold is the highest possible value of spacing, such that its frequency is higher than both 10 and double the frequency of the spacing one unit above, and such that all lower values of spacing have higher frequencies. However, if there is a higher value of spacing with a frequency above 100, this value is used as a threshold instead. This is summarized in the formula that follows:
  $$\begin{array}{ccc} threshold & = & \max\{s|s\in spacing\;\wedge \;\\ & & \;[\;(\;frequency(s)\;\geq \;10\;)\;\wedge \\ & & \;(\;frequency(s)\;\geq \;(2\;*\;frequency(s+1))\;)\;\wedge \\ & & \;(\forall \;t\;\in \;spacing\;:frequency(t)\;>\;frequency(s))\;]\\ & & \;\vee frequency(s)\;>\;100\} \end{array}$$ (1)
  For “Harry Potter and the Philosopher’s Stone” [1], the computed value is 8 (i.e. If more than eight lines do separate two dialogs, they do belong to two different conversations), as shown on Fig 8. It is then used to group the dialogs and to single out the successive conversations appearing in the novel.
3. **Context generation:** To improve the process of speaker identification coming later on during the process, the conversation is being extended to a context. (i.e. The named entities found inside the corresponding context are used to enrich the information already extracted from the dialogs forming a conversation) It is built by listing every sentence from the end of the previous conversation up to the start of the next one. (See Fig 3)
4. **Dialog metadata extraction:** For each dialog found inside the novel some metadata are computed and grouped to facilitate further processing. The result is a map of every dialog associated with the index of the dialog (i.e. the index of the corresponding line inside the novel, indicating the time at which the dialog occurs) as well as two fields, ‘from’ and ‘to’, containing the names identified and corresponding respectively to the speaker and the audience. Here they are explained with an example in Fig 9.

**Metadata**:

- **Index of the sentence:** Position of the line of dialog inside the entire text.
- **Identified speakers (‘from’):** Character that speaks at that moment.
- **List of the identified characters within the dialog (‘to’):** Characters mentioned in the dialog. This list contains all the NNPs (proper nouns) identified in it.
- **Index of the context to which that dialog belongs:** Corresponding to the number of the context (i.e. enriched conversation) in which the dialog falls after the conversation thresholding.

At the end of this step, dialogs are grouped into conversations, each of which has an associated context, and each dialog is enriched with metadata that serves as the basis to build a social network.

### Characters identification and alias resolution

Another problem occurring while reading a novel is the speaker and characters identification. “Who talks to whom?” illustrates the problem perfectly. Once again, this task is easy for a human but may be more difficult for a program depending on the writing style of the author (Namely, whether the book is intended for a young or an adult readership). Identifying the characters involved in the action and more specifically distinguishing the speaker from the audience is another difficult problem to solve.

This part of the algorithm runs in two steps and relies on the metadata previously computed (i.e. the parse tree and chunks already provide the information about the NNPs that are looked for) (See Table 1).

**Fig 10:**
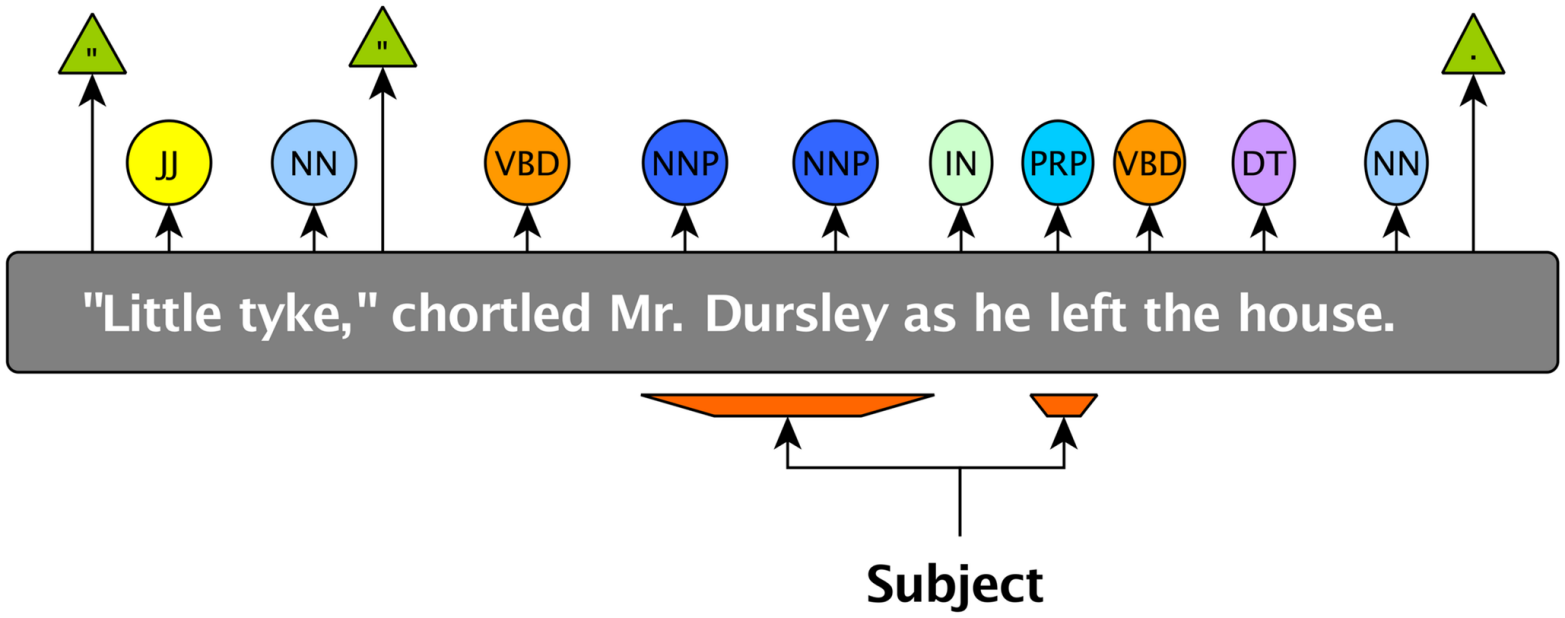
Example of the first step of the speaker’s identification process. Locating the subject of the sentence.

1. **First**, the program takes each dialog of the novel and checks its grammatical structure computed while pre-processing the novel. During the metadata extraction in the previous step, the program already checked for the presence of dialog tags (bits of narration interspersed within the dialog to indicate a speaker, e.g. “he said”) and extracted a speaker if the subject of that dialog tag was a proper noun (NNP tag), or a succession of proper nouns and stores it as the “from” field of the dialog occurrence. Otherwise, the speaker is not yet clearly identified, and that field is blank. (See Fig 10 for an example and section for more information).
2. **Second**, the problem of aliases is handled. Almost every characters possesses one or more aliases used through the story telling by the author at different moments (i.e. name, diminutive, title, full name,…) This step handles this problem by building an alias network which provides an association of the different names with their aliases and replaces their occurrences by the same name; the key of the character. Doing so, instead of risking to create a network full of characters different in appearance but actually identical, the algorithm is able to reduce the size of the resulting network by properly identifying the characters.

**Table 1:** Part-of-speech tags.

**Tag**	**Meaning**
CC	Conjunction, Coordination
CD	Cardinal number
DT	Determiner
EC	Existential there
FW	Foreign word
IN	Conjunction, subordinating proposition
JJ, JJR, JJS	Adjective (regular, comparative and superlative)
LS	List item marker
MD	Verb, modal auxiliary
NN, NNS, NNP, NNPS	Noun (singular or mass, plural, proper singular, proper plural)
PDT	Predeterminer
POS	Possessive ending
PRP, PRP$	Pronoun (personal, possessive)
RB,RBR,RBS,RP	Adverb (regular, comparative, superlative, particle)
SYM	Symbol
TO	Infinitival to
UH	Interjection
VB, VBZ, VBP, VBD, VBN, VBG	Verb (base form, 3rd person singular present, non-3rd person singular present, past tense, past participle, gerund or present participle)
WDT, WP, WP$, WRB	Wh-words (determiner, personal pronoun, possessive pronoun, adverb)
. ,: ()	Punctuation marks

Two main flaws arise from the use of the alias table however: the inability to identify characters from other proper nouns, and the handling of NNP tags that are applied to several characters, such as titles and family names.

The part-of-speech tags are based on the Brown Corpus [59] reduced in size to fit the orientation of the Penn Treebank [61, 62] used. It eliminates redundancy by taking into account lexical as well as syntactic information. Most NLP tools use those.

The former problem derives from the use of the Pen Treebank [61, 62]) tag set as the primary identifier of character names. Indeed, a variety of proper nouns are tagged as NNPs (Example: Gryffindor) with no regard to what they refer to. Because of this, we are unable to differentiate capitalized neologisms or named locations from character names.

The second error comes from titles and family names when several characters share them. It is impossible to fully solve those aliases, but a workaround (the alias table) was implemented to prevent those names from being referred to as mush as possible (e.g. “Professor McGonagall” would be included in the entry “McGonagall” but not professor, but if the author refers to her used only her title then the node “Professor” would still appear).

It should also be noted that the algorithm is incapable of differentiating between two characters who would have the same name. This could be corrected by employing a disambiguation algorithm; however, that only results to minor errors: characters in a fictional narrative, especially in writing, are rarely named the same way [63], as that could cause confusion even for a normal reader. This is consistent with other research in stylistic analysis over works of fiction, such as Argamon et al. [64]. It should be noted that a disambiguation algorithm may help the second error mentioned above; this may thus be the subject of future research.

**Speaker identification**. During the metadata extraction step, speakers are identified locally for each dialog (i.e. within the dialog itself), using dialog tags as announced. This means that the resulting speaker identification rate is highly dependent on the author’s style; namely, whether or not they frequently remind the reader of who is speaking, and whether or not they use the character’s name or pronouns to do so.

The resulting rate of speaker identification (SIR), averaged over all analysed books, is close to 50%, but with a high variance (standard deviation of about 10%). A breakdown by author, however, shows the expected result: the average rate of identification is very different depending on the writer. (See Table 2)

**Table 2:** Rate of speaker identification (SIR).

**Author**	**Average SIR**	**Std dev**
C. Clare	50.04%	3.2%
R. Hobb	50.46%	3.5%
V. Hugo	37.85%	N/A
R. Jordan	64.65%	5.5%
R. J. & B. Sanderson	52.95%	1.01%
G.R.R Martin	48.08%	4.41%
M. Meyer	33.81%	0.86%
P.Pullman	40.91%	4.84%
J.K. Rowling	57.27%	3.55%

For V. Hugo the standard deviation could not be computed since only one book was considered.

For B. Sanderson, due to the last three *Wheel of Time* books being written by a different author, they have to be considered separately.

This leads to an acceptable rate of speakers identification. To improve the results obtained, some assumptions are made. For example, in a conversation where only two characters were identified as speakers, the algorithm infers that the characters speak in turn, and fits that pattern to the dialogs where no speaker has been identified yet as best as possible. The same happens when more than two characters are involved in a conversation: the algorithm takes into account the speakers identified for the neighboring dialogs to propose the best possible inferences. Doing so, the average rate of speaker identification reaches over 98%.

One question arises though: what about the accuracy of this identification? At this stage of development the accuracy cannot be measured for a simple reason: the correct speakers should be identified manually to assess the precision of the classifier. As an example, in *Harry Potter and the Philosopher’s Stone* [1], the correct speaker was identified for 56.9% of individual occurrences.

**Fig 11:**
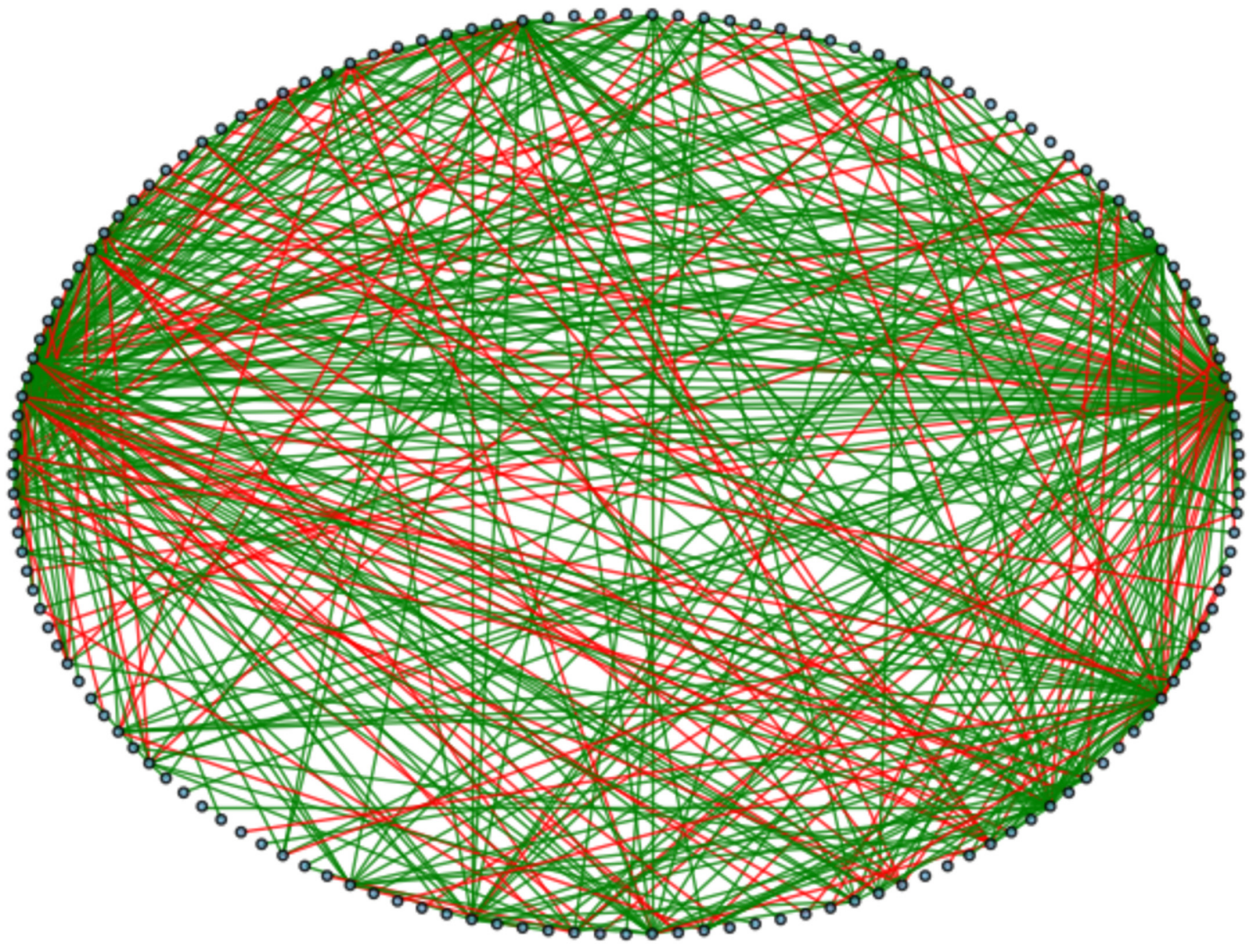
Differential between the extracted and correct networks in “Harry Potter and the Philosopher’s Stone” [1]. Red edges are incorrect edges, green edges were identified correctly.

However, the bulk of the analysis does not rely on individual occurrences, but on networks built from entire conversations. Within each conversation, all the identified speakers are interconnected; thus, as long as a speaker was identified within a conversation, it matters little whether or not it was assigned the correct specific lines of dialog, as it will result in the same network. This is measured by comparing the list of speakers appearing in each context of the extracted network to the same list in each context of a manually-generated correct graph, and counting how many are present in both lists. For the example of *Philosopher’s Stone*, the proportion of speakers that were correctly found goes up to 82.4% when performing this bulk analysis. To get a better sense of what this represents, a differential between the extracted graph and the correct graph is shown in Fig 11. 73.3% of the edges from the extracted are present in the correct graphs.

The discrepancy can be explained by some of the assumptions made, especially when assuming a strict order in which characters speak within a conversation. This means that any error propagates quickly within a conversation, for instance by “flipping” the speakers, whereas the characters identified for that conversation might remain correct.

To synthesize, this step is the most important. It tries to identify all the characters as well as their aliases and allows the identification of a speaker for a majority of the dialogs encountered. However, the interest of the speaker identification may seem useless at first glance. Indeed, building a social network should lead in a first approximation to an undirected network in which all the characters that do present social links are linked together. The speaker identification allows the algorithm to go further by giving a direction to each link, providing for a directed, dynamic network with an edge weight corresponding, for example, to the sentiment transmitted by the speaker. This aspect goes however out of the bounds of this paper and will be the object of later analysis. Here, the capabilities of the algorithm are just demonstrated.

### Network building

The final step of the process consists in the creation of the network. It is the simplest part in principle, but the potential outputs are numerous. Do we want a simple social network (undirected, unweighted, atemporal), do we want the network to be extracted from only one chapter, one context, one book or even an entire series? Do we want it to be dynamic? With sentiment scores? Do we want the scores obtained to be adapted over time or not? Many other networks can also be extracted such as the alias networks, dialog networks… However, for this paper, only social networks are used. Two types of social networks are extracted during the processing, each giving a different characteristic: the “Context Networks” and the “Incremental Networks”.

**Fig 12:**
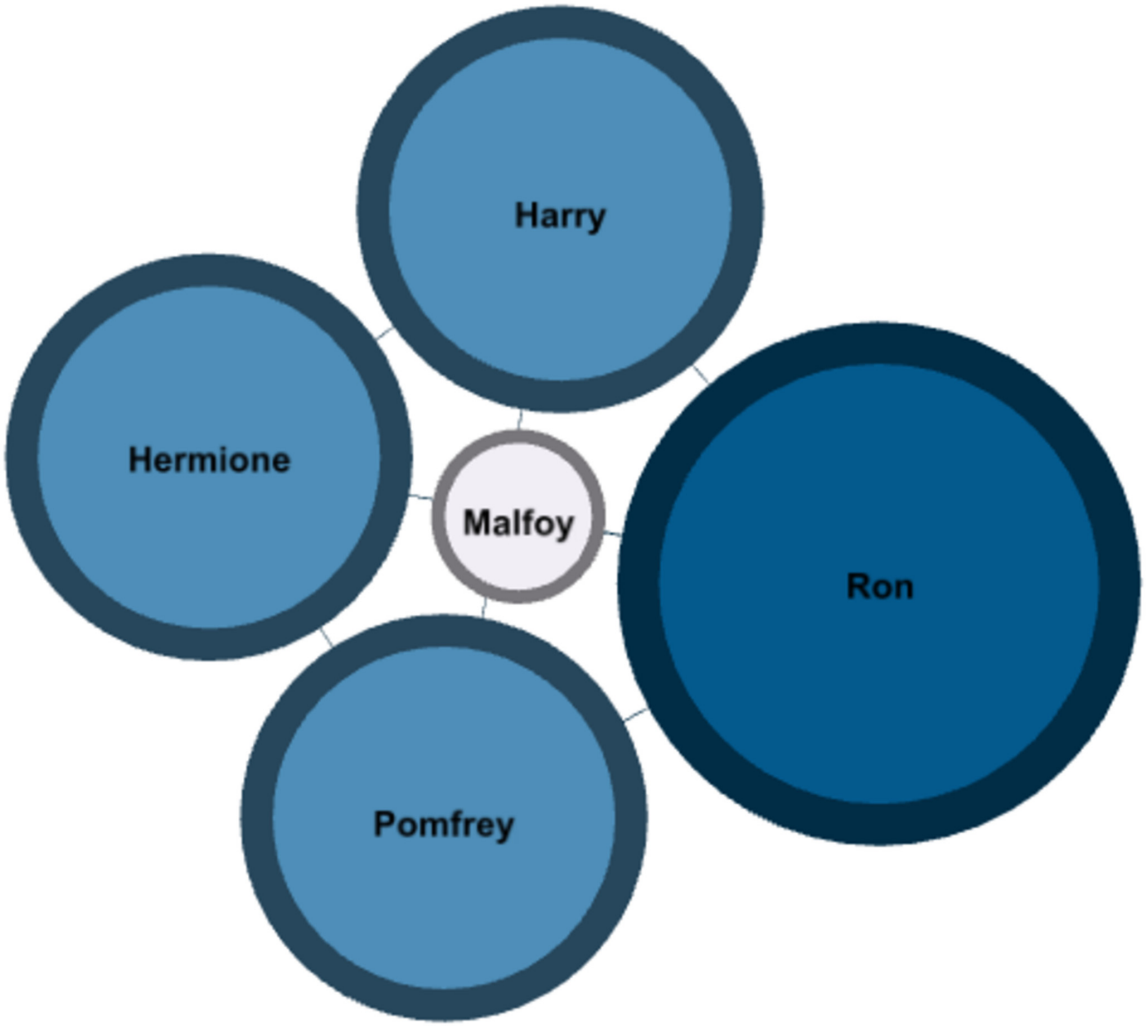
Context network #64 in “Harry Potter and the Philosopher’s Stone” [1].

The **“Context Networks”** are built using only the dialogs corresponding to a single conversation, for which the network is built. The resulting graph is undirected, since the interactions are considered to be reciprocal between two characters. All characters appearing in the context are interconnected. This is not mandatory: we could identify the speaker and the audience for each dialog and build a directed graph based on that information. Those networks can be used to analyse a precise sequence of storytelling, but are not the ones on which the following results are based; they will be the subject of further research. An example is given in Fig 12 for the context number 64 in the first book of Harry Potter.

**Fig 13:**
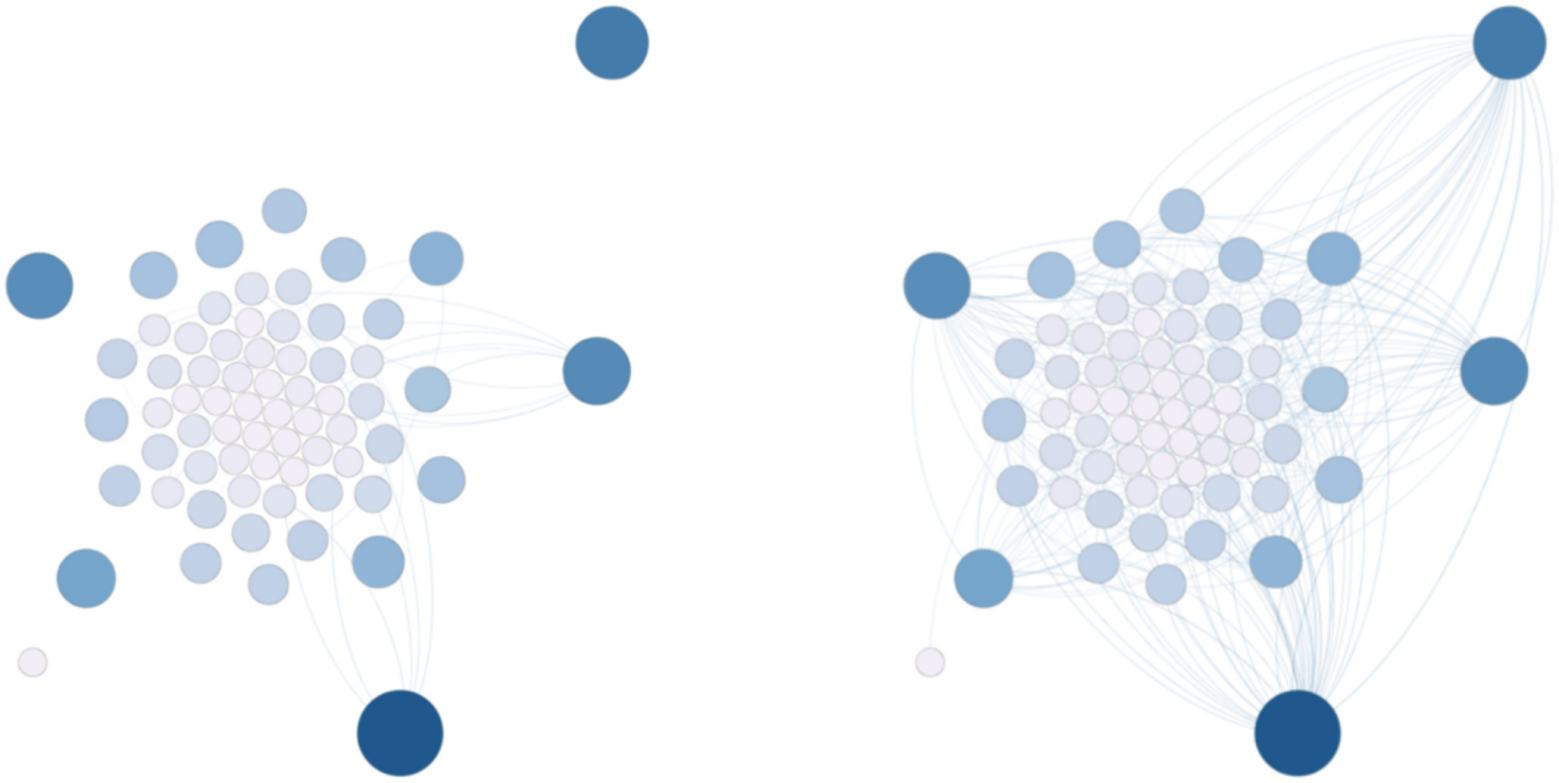
Example of incremental networks for “Harry Potter and the Philosopher’s Stone” [1]. On the left, context 0 to 498. On the right, context 0 to 1670.

The **“Incremental Networks”**, on the other hand, are built iteratively from the context networks by merging all the context network until some point in the story; in other words, by taking all context networks from the first conversation to a given conversation, and merging them together. An example is given in Fig 13 with 498 contexts considered on the left side (Fig 13 left), roughly corresponding to a specific period of the novel in which only the family members of the main character, Harry, are interacting, as well as a few professors; and the global network on the right side (Fig 13 right), built using the 1670 dialogs available.

**Fig 14:**
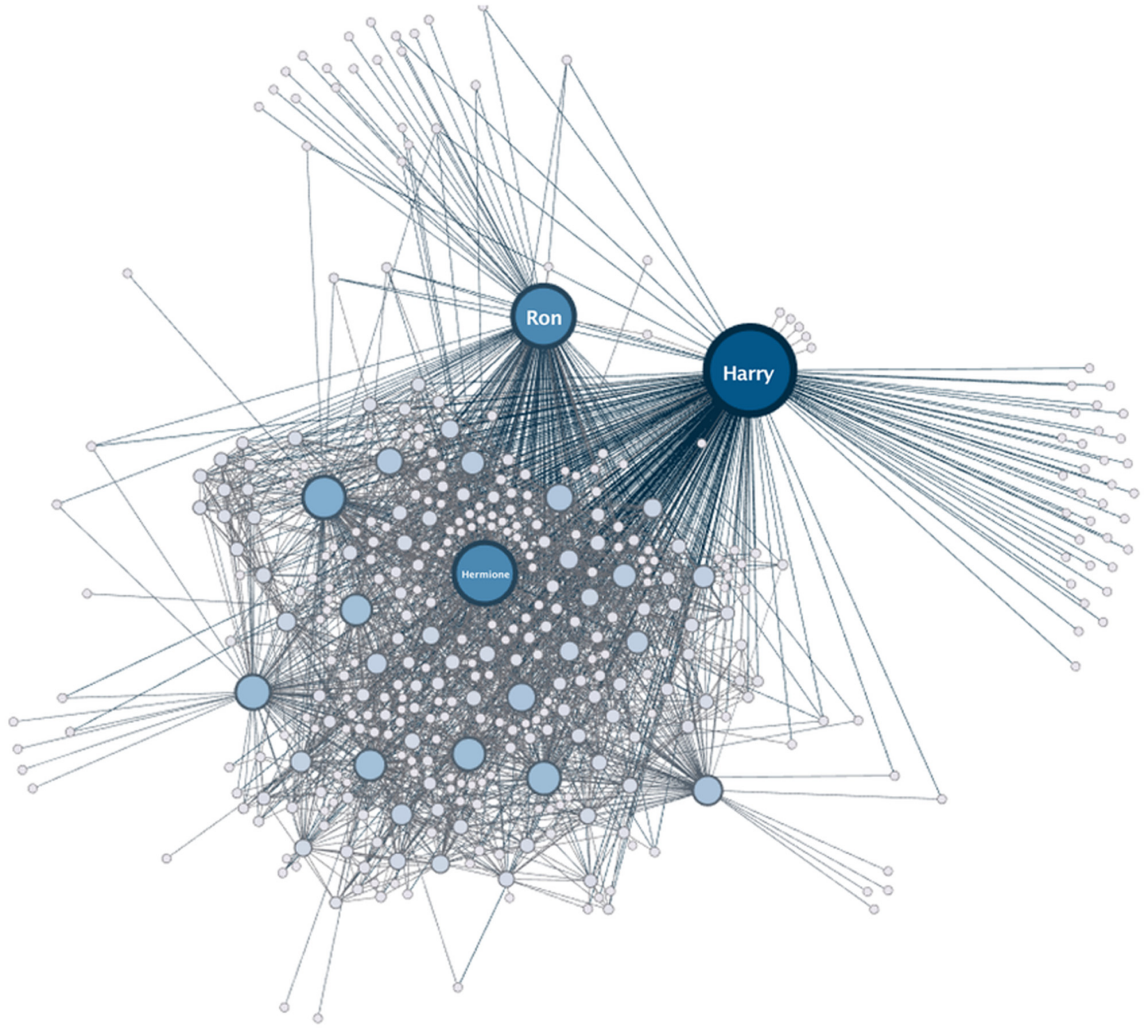
Network Extracted from the book “Harry Potter and the Order of the Phoenix” [5].

Every network is exported to the CSV format and drawn as a PNG image, while the final incremental graph, representing the social network at the end of the story, is written to the additional formats GEXF and JSON. The possibilities of outputs are obviously numerous and will undergo additional research in the future. Fig 14 shows an example of such a final graph from the Harry Potter series.

In summary, the algorithmic steps just described are able to generate many different types of networks all answering to specific questions. In this paper, we have decided to restrict our analysis on one main aspect: the topology and the time evolution of the characters social network (preferential attachment or not) as well as how they do compare with their real counterparts.

## Results

As outcome of the execution of the algorithm many results can be obtained. For the purpose of this article, mainly one example resulting from the social network extraction on “Harry Potter” is examined more deeply, even though the other books and series were analyzed.

### Degree distribution

**Fig 15:**
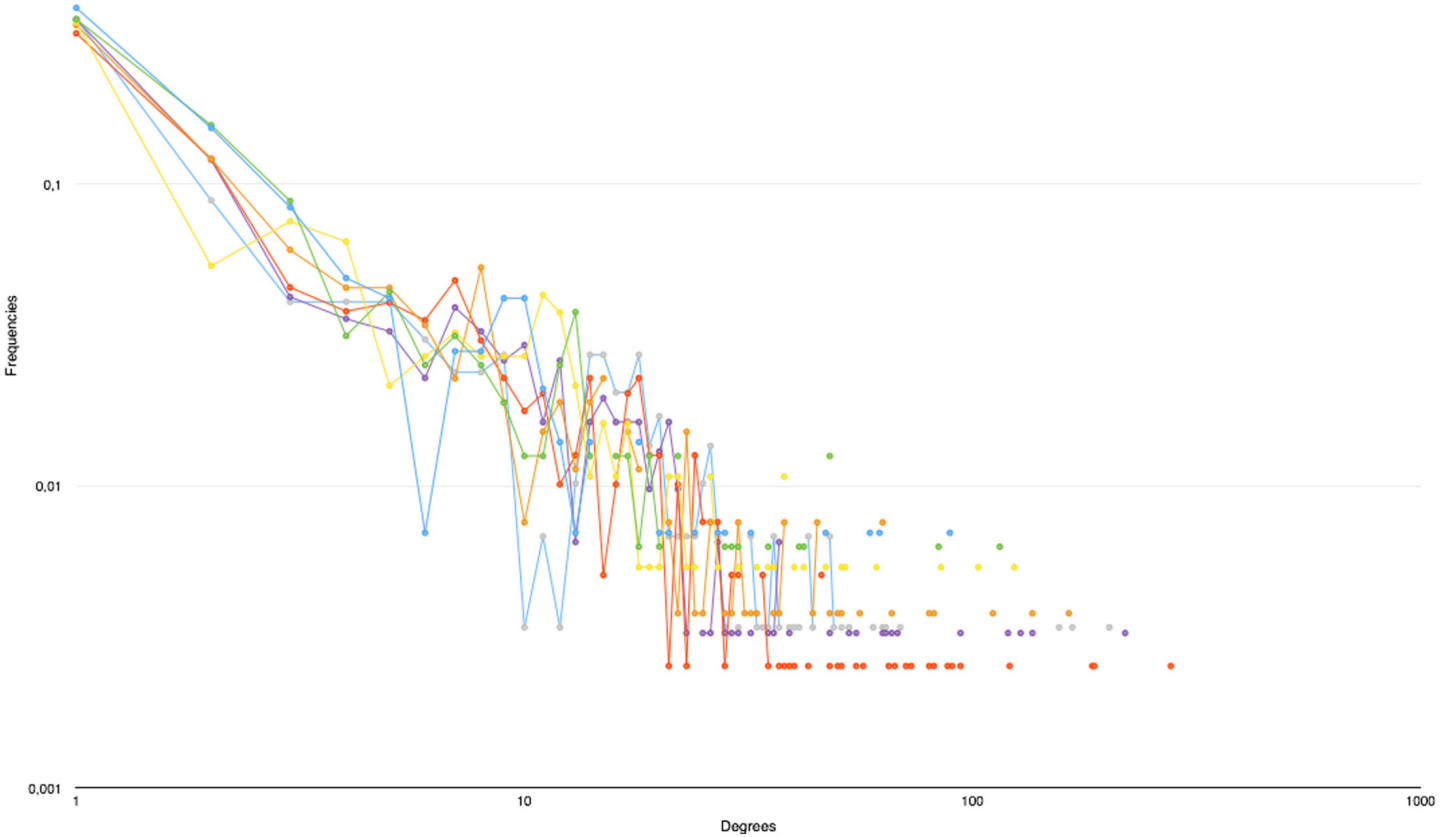
Degree distribution for all Harry Potter books.

The degree distributions for each book (See Fig 15 for the distributions of the book from the Harry Potter series) show similar profile and appear similar to the typical distribution of a scale-free network.

It should be noted that, while there is a discrepancy with an ideal power law, it is fairly small (as indicated by the *R* ^2^ of the power function fitted to each distribuion) and mostly due to the small size of the networks, meaning that low frequencies for the highest degrees cannot effectively be reached. (See Table 3) The exponent of the power approximation falls in the [−1, −2] range, which is outside of the [−2, −3] predicted by theory [49] or shown by large, scale-free networks, such as the Web graph [65]. However, social networks do show exponents falling in that range ([66–69]). That the networks extracted from novels fall into the lower end of that range can be attributed to two factors that contribute to increasing the probabilities of higher degrees:

- Conversations tend to involve several people at once, either due to several people participating in a conversation or because another character is mentioned during the exchange.
- The specificities of fiction, in particular the so-called “Law of Conservation of Detail” [70], which dictates that any element mentioned in a work of fiction should be relevant (and thus be brought back up again).

**Table 3:** Exponent of the power approximation.

**Book**	**Equation**	***R*^2^**
Harry Potter 1	*y* = 0.3166*x* ^−1.2492^	0.8463
Harry Potter 2	*y* = 0.2785*x* ^−1.1547^	0.8888
Harry Potter 3	*y* = 0.2320*x* ^−1.0662^	0.8586
Harry Potter 4	*y* = 0.2668*x* ^−1.1720^	0.8722
Harry Potter 5	*y* = 0.3177*x* ^−1.2498^	0.8836
Harry Potter 6	*y* = 0.3088*x* ^−1.2306^	0.8816
Harry Potter 7	*y* = 0.1994*x* ^−1.0549^	0.7682
A Game of Thrones	*y* = 0.3585*x* ^−1.2720^	0.8723
A Clash of Kings	*y* = 0.3032*x* ^−1.2195^	0.8455
A Storm of Swords	*y* = 0.3985*x* ^−1.3360^	0.9123
A Feast of Crows	*y* = 0.2713*x* ^−1.1743^	0.8289
A Dance with Dragons	*y* = 0.3207*x* ^−1.2020^	0.8607
Nothern Lights	*y* = 0.3685*x* ^−1.3620^	0.8951
The Subtle Knife	*y* = 0.4106*x* ^−1.4232^	0.8952
The Amber Spyglass	*y* = 0.2782*x* ^−1.1661^	0.8238
Les Misérables	*y* = 0.3441*x* ^−1.5805^	0.9291

**Fig 16:**
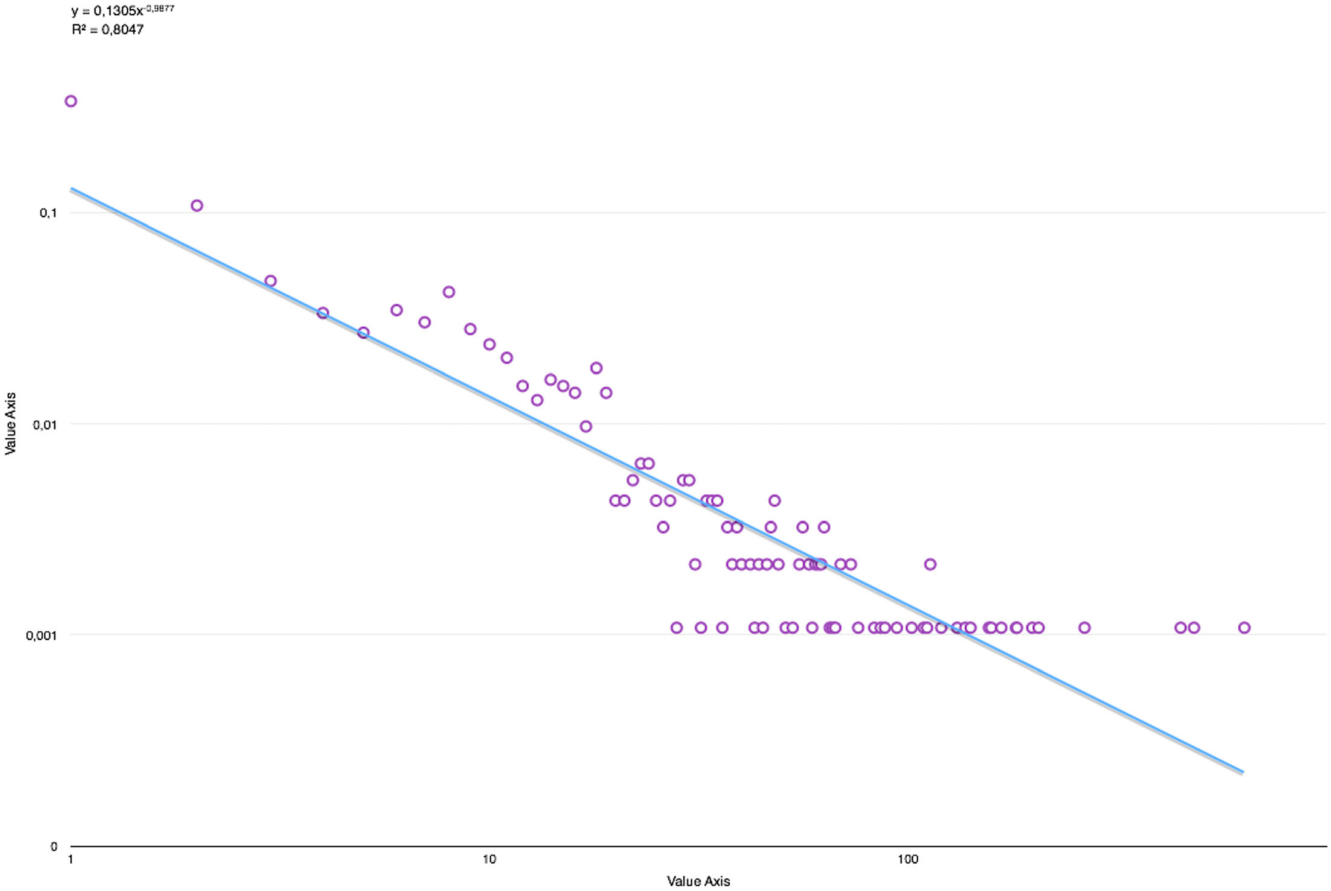
Degree distribution in the full “Harry Potter” series plus the power approximation.

These factors, added to the networks’ small size, contribute to increasing the real frequencies for higher degrees. This is true, even when considering a series as a single entity, as shown on Fig 16.

### Clustering coefficient

**Fig 17:**
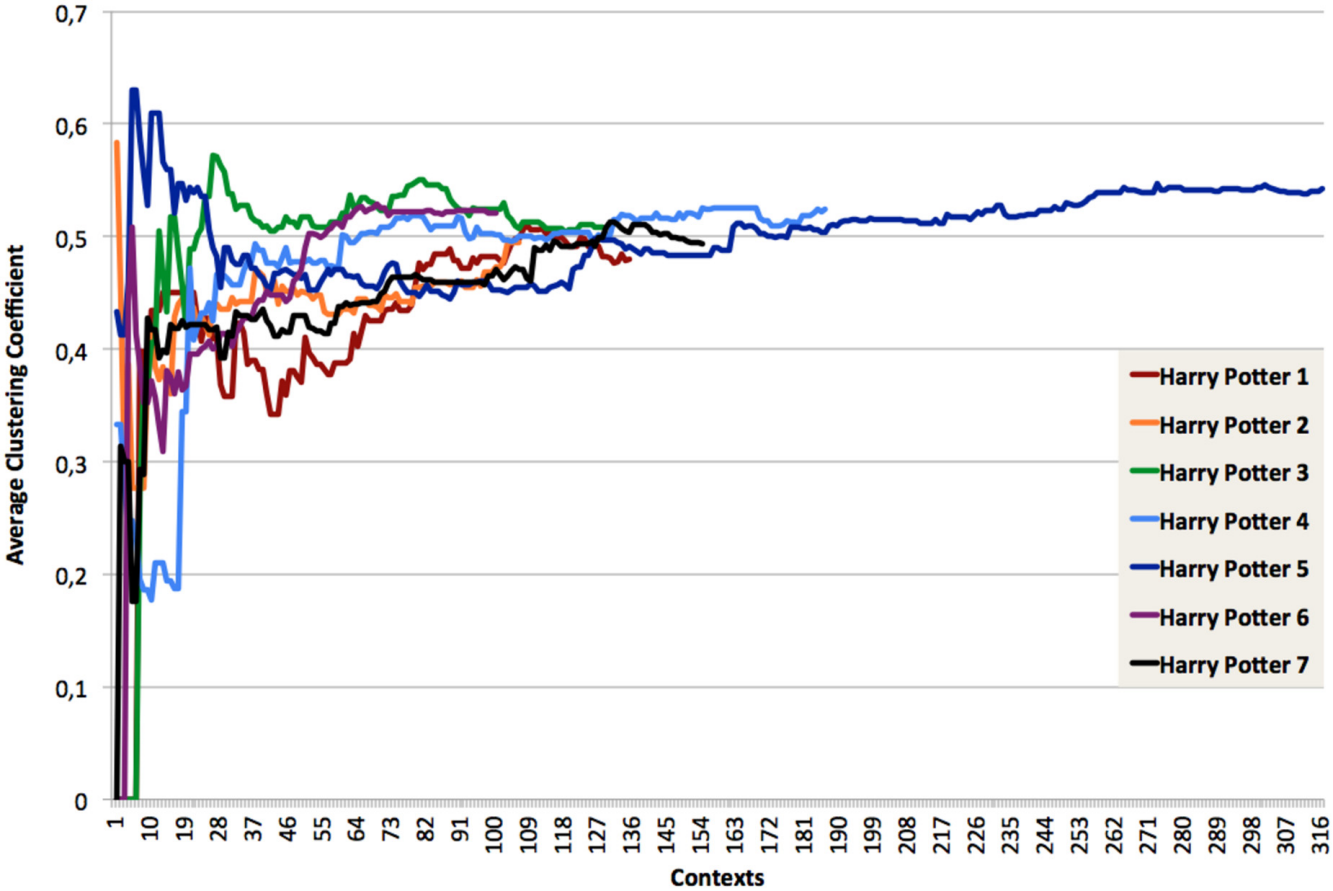
Clustering coefficient in the Harry Potter books.

**Fig 18:**
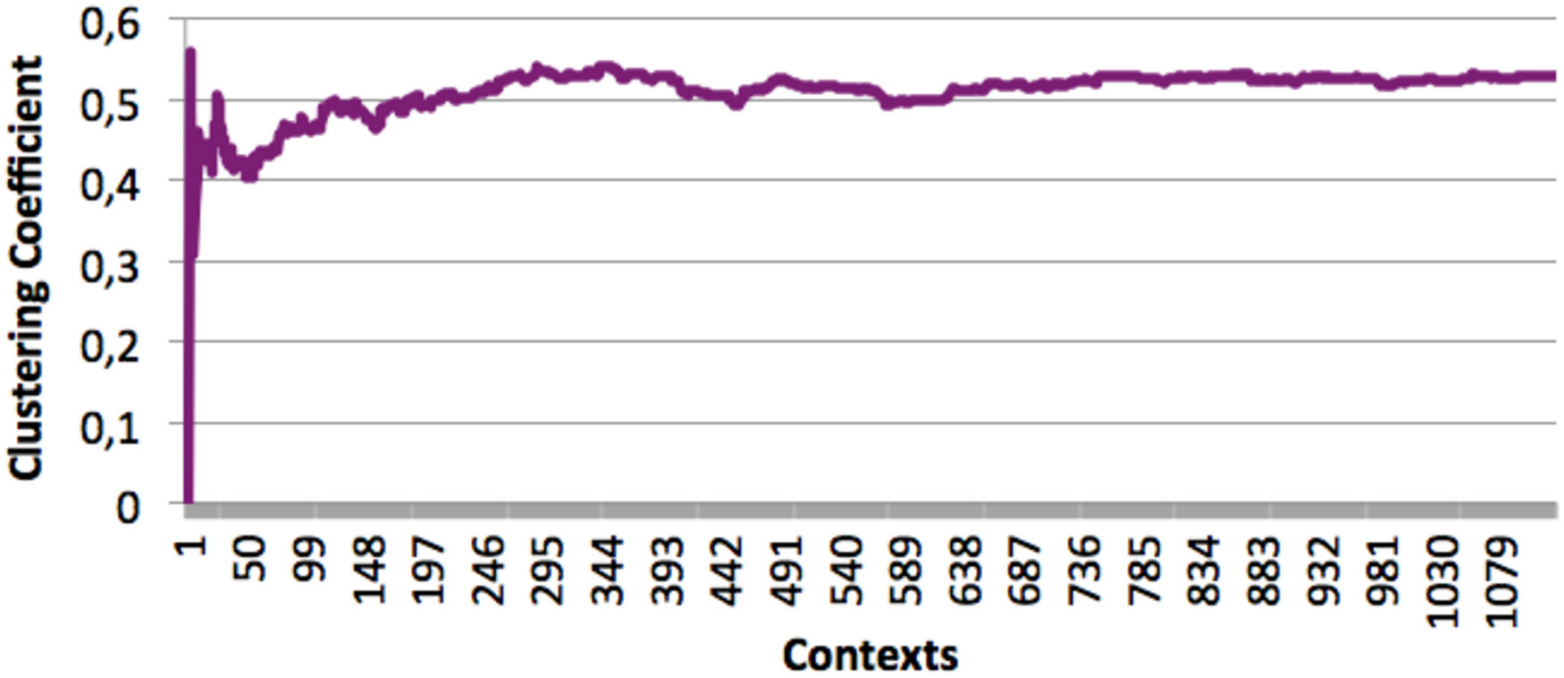
Average clustering coefficient for the seven “Harry Potter” books processed as a single entity.

The clustering coefficients of the incremental networks produced were also computed (See Figs 17 and 18). It shows a clear common trend for all the books: the clustering coefficient starts at a value which is heavily dependent on the first few contexts occurring in the book to quickly stabilize at a value close to its final one. The values then converges slowly.

To assess the significance of the clustering coefficient in a rigorous way, the best point of comparison to use is a similar random graph, whose clustering coefficient is given by *C*_*rand*_ = < *k* > /*N*. The same values are computed for the extracted social networks, i.e. their **average clustering coefficient** [48]. The coefficient of a random graph is the proportion of two neighbouring nodes that are connected, equivalent to the probability that two random nodes are connected. The comparison is presented in the table from Fig 4.

The order of magnitude of the coefficient is greater for all the books considered, the same type of results that characterizes scale-free networks, as shown for instance in [48, 66, 67]: one of the common characteristic of social networks [73] is a high ratio of their clustering coefficient to that of a random graph with similar properties (average degree and size).

Going further in the analysis, the clustering coefficient can be explained with more detail by studying the type of narration of each book. For instance, a book that focuses on a single character or group of characters will present a higher clustering than stories focused on several characters. Similarly, extending the scope of the story across time or space will have a tendency to reduce the resulting social network’s clustering.

For instance, compare two very different series: *Harry Potter* and *A Song of Ice and Fire*. The former, focused on a single protagonist, features a much higher clustering coefficient than the latter, in which the story follows several protagonist, often very separated in-universe. Another example is within a single series itself, *The Wheel of Time*: the clustering coefficient changes greatly from books where the protagonists are few and/or gathered for a single event (“New Spring”, “The Eye of the World”, “Winter’s Heart”) to when the characters are very separated (“The Dragon Reborn”, “The Gathering Storm”). (See Table 4)

**Table 4:** Average clustering coefficient for each book & clustering coefficient for a similar random network.

**Book**	**Clustering**	**Random**
Harry Potter 1	48.00%	4.47%
Harry Potter 2	49.43%	4.75%
Harry Potter 3	50.80%	5.39%
Harry Potter 4	52.37%	3.92%
Harry Potter 5	54.19%	2.92%
Harry Potter 6	52.07%	3.32%
Harry Potter 7	49.40%	3.92%
A Game of Thrones	48.35%	2.95%
A Clash of Kings	47.48%	2.58%
A Storm of Swords	45.23%	1.82%
A Feast of Crows	51.51%	2.29%
A Dance with Dragons	53.24%	2.62%
Nothern Lights	35.41%	3.48%
The Subtle Knife	43.29%	4.07%
The Amber Spyglass	34.55%	4.74%
Les Misérables	20.55%	0.61%
Cinder	33.16%	6.43%
Scarlet	52.38%	5.93%
Cress	42.16%	5.65%
Ship of Magic	46.17%	2.69%
The Mad Ship	35.22%	2.99%
Ship of Destiny	45.82%	4.29%
The Dragon Keeper	33.44%	4.43%
Dragon Haven	36.5%	4.83%
City of Dragons	25.14%	3.35%
Blood of Dragons	33.29%	4.98%
City of Bones	47.76%	4.01%
City of Ashes	45.81%	4.93%
City of Glass	42.85%	4.74%
City of Fallen Angels	43.16%	3.80%
City of Lost Souls	42.61%	3.04%
City of Heavenly Fire	42.78%	3.88%
New Spring	49.62%	4.54%
The Eye of the World	49.35%	2.80%
The Great Hunt	41.08%	2.67%
The Dragon Reborn	37.23%	2.22%
The Shadow Rising	45.16%	2.34%
The Fires of Heaven	38.9%	2.29%
Lord of Chaos	38.87%	1.67%
A Crown of Swords	38.21%	1.79%
The Path of Daggers	35.32%	1.74%
Winter’s Heart	42.10%	2.29%
Crossroads of Twilight	44.4%	2.21%
Knife of Dreams	39.11%	1.35%
The Gathering Storm	34.36%	1.80%
Towers of Midnight	37.04%	1.49%
A Memory of Light	42.37%	2.17%

### Preferential attachment

**Fig 19:**
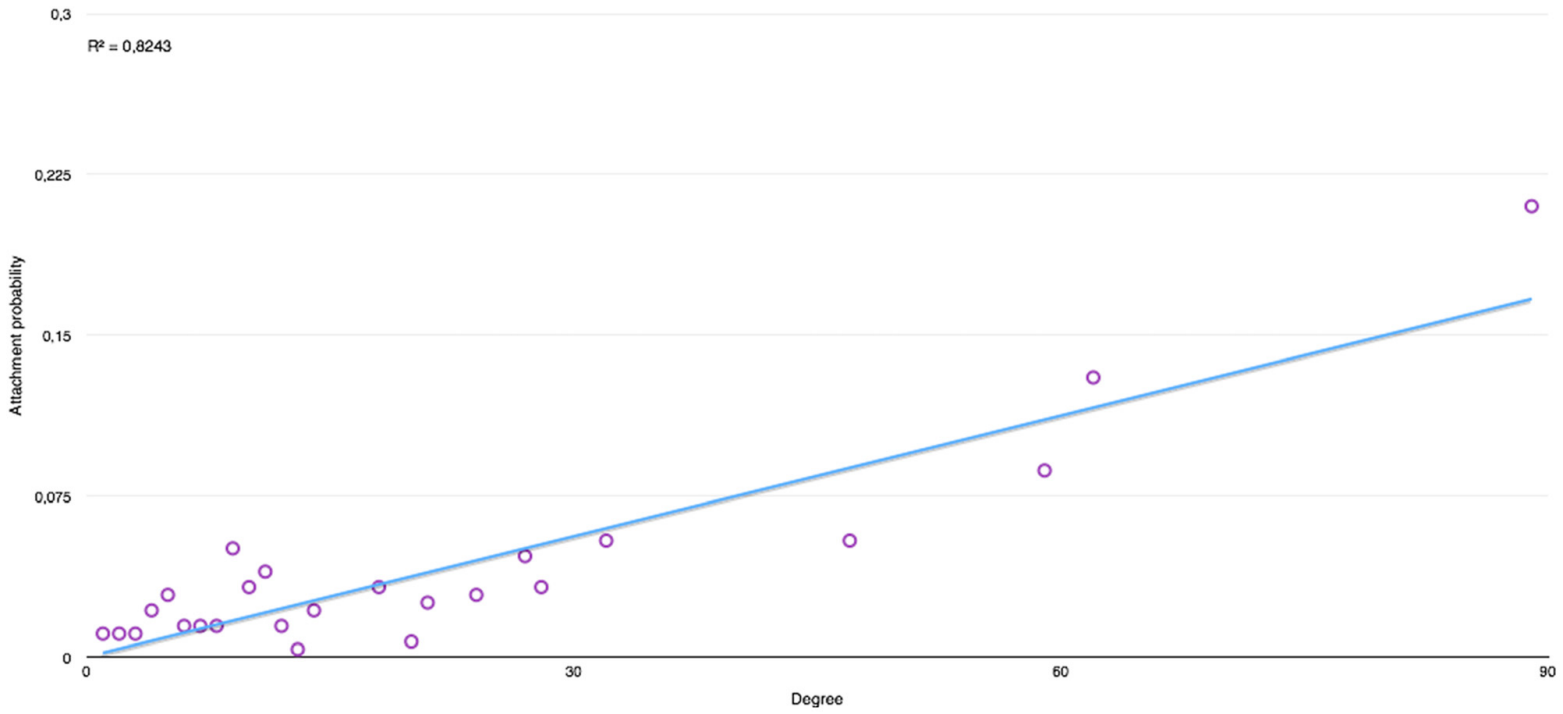
Preferential attachment in “Harry Potter and The Philosopher’s Stone” [1]. This plot represents the probability, given the degree of a given node at a given time, that a new node being added to the social network will have a connection to that node.

**Fig 20:**
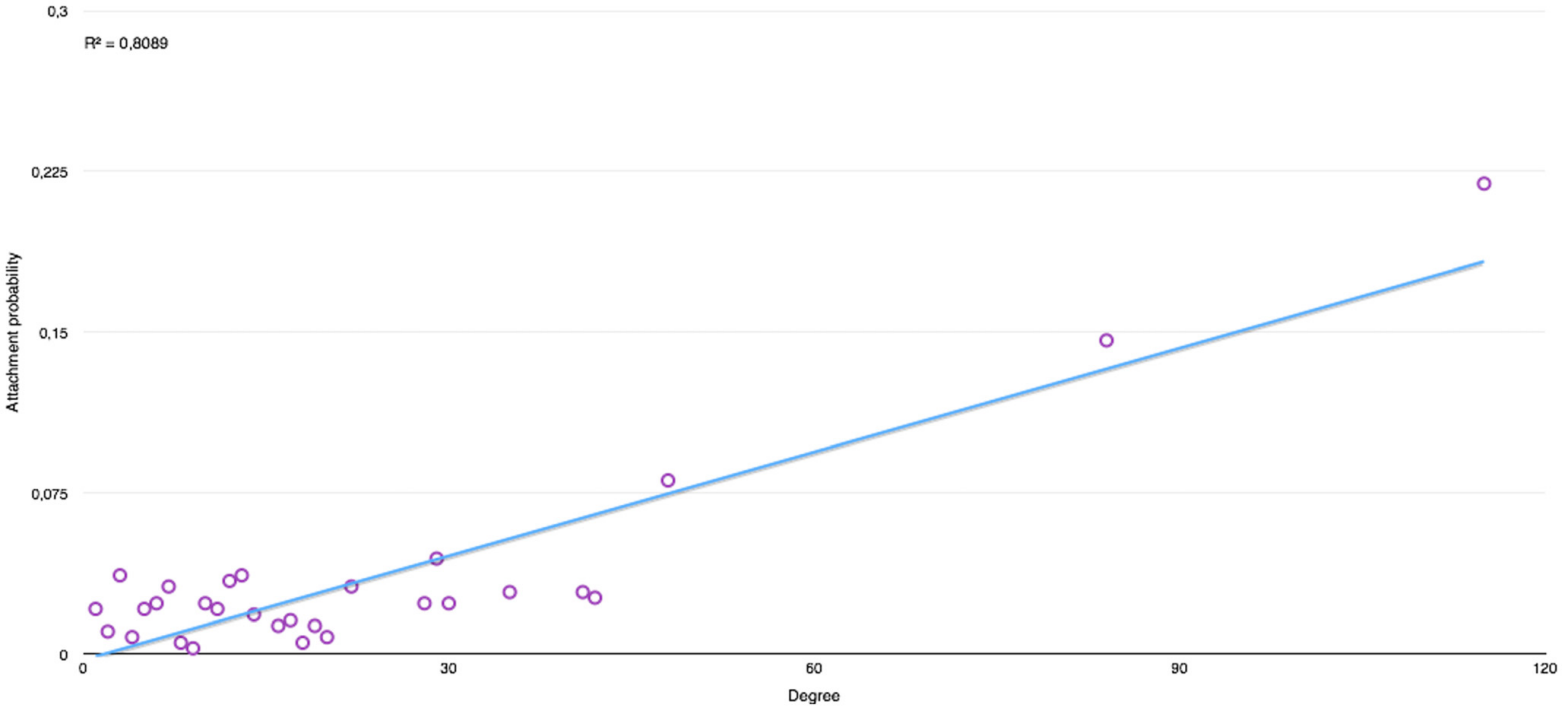
Preferential attachment in “Harry Potter and The Chamber of Secrets” [2].

Preferential attachment has also been measured and evaluated. The plots in Figs 19 and 20 represents the probability that a new node be attached to a node of a given degree, with those degrees being taken a posteriori from the global graph. Those profiles are tested by comparing them to a fitted linear function.

From those graphs, we can observe that books with a single protagonist have a much higher tendency towards preferential attachment. Indeed, we can verify that in other series like “His Dark Materials”, the *R* ^2^ values goes down tremendously due to the introduction of a new protagonist in every new book, where the value of *R* ^2^ remains similar in the series of “Harry Potter” as well as in the other books.

### Clustering of the books

**Fig 21:**
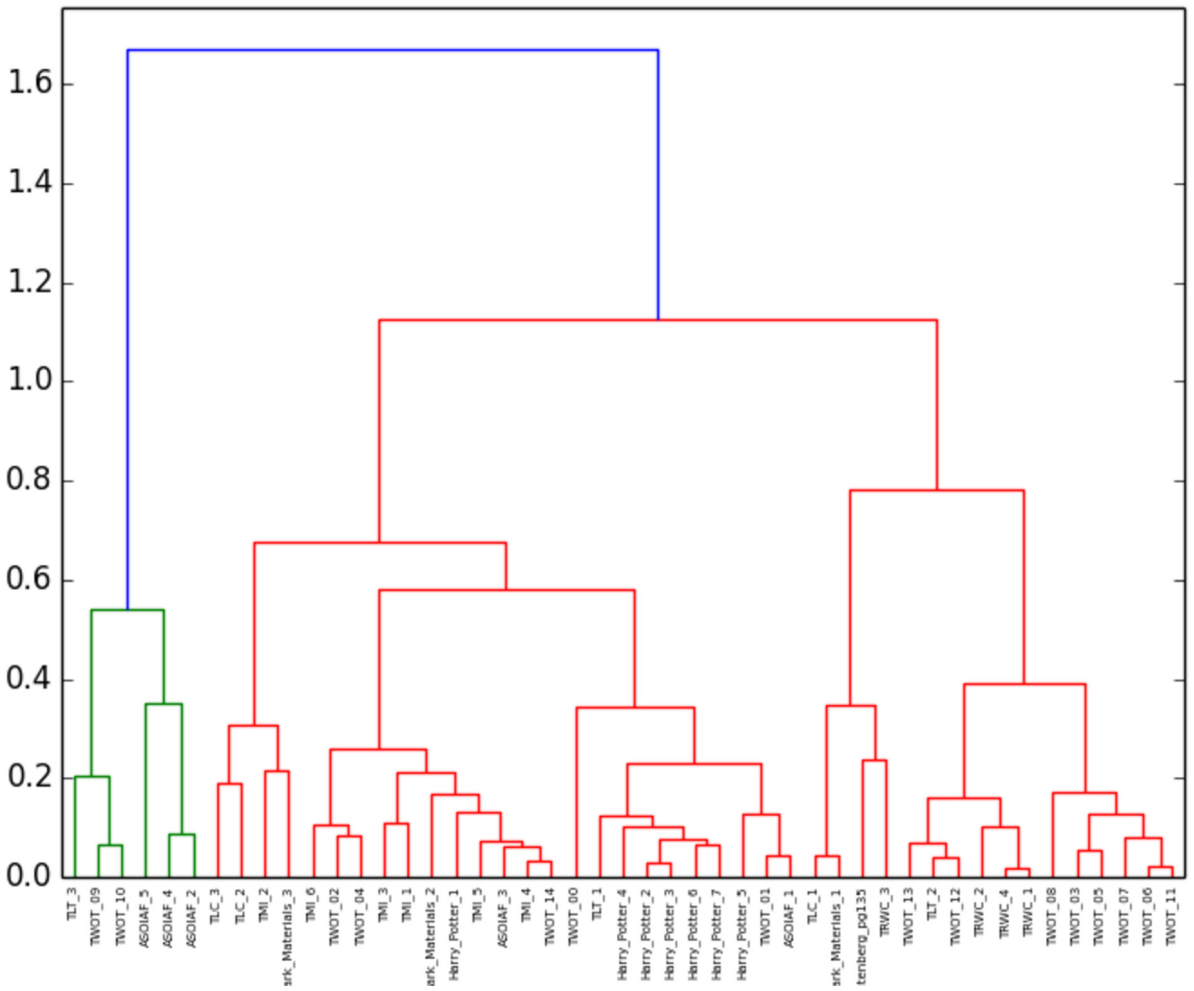
Dendrogram for the hierarchical clustering of the books using the Ward variance minimization algorithm.

During the extraction of each book’s social network, a number of signature features were identified: the conversation size threshold, the speaker identification rate, the degree distribution’s exponent, and the average clustering coefficient in the final network. Two methods of clustering were implemented to test these four features: hierarchical clustering, using the Ward variance minimization algorithm, on Fig 21, and a K-means classification, shown in Table 5.

**Table 5:** Clusters generated with K-Means, K = 10.

1	‘His Dark Materials 3’
2	‘TLT 2’, ‘TRWC 4’, ‘TRWC 3’, ‘TRWC 2’, ‘TRWC 1’, ‘TWOT 13’, ‘TWOT 12’
3	‘Les Miserables’, ‘TLC 1’, ‘His Dark Materials 1’
4	‘TLC 3’, ‘TLC 2’, ‘TMI 2’
5	‘TWOT 01’, ‘TWOT 04’, ‘ASOIAF 1’, ‘TLT 1’, ‘Harry Potter 1’, ‘Harry Potter 2’, ‘Harry Potter 3’, ‘Harry Potter 4’, ‘Harry Potter 5’, ‘Harry Potter 6’, ‘Harry Potter 7’
6	‘ASOIAF 4’, ‘ASOIAF 5’, ‘ASOIAF 2’, ‘TLT 3’
7	‘ASOIAF 3’, ‘TMI 4’, ‘TMI 5’, ‘TMI 3’, ‘TMI 1’, ‘His Dark Materials 2’, ‘TWOT 14’
8	‘TWOT 08’, ‘TWOT 02’, ‘TWOT 03’, ‘TWOT 06’, ‘TWOT 07’, ‘TWOT 05’, ‘TMI 6’, ‘TWOT 11’
9	‘TWOT 09’, ‘TWOT 00’, ‘TWOT 10’

The dendrogram shows a good clustering of certain series: “Harry Potter”, “The Rain Wild Chronicles”, “The Lunar Chronicles” and “The Mortal Instruments”, for instance, appear to be clustered. Other series are more scattered, which can be attributed to a fluctuation of style: for instance, “The Wheel of Time” is separated in three sub-clusters, gathering similar books within the series together.

The K-means classification yields better results: although some series are still split across several clusters, they are much less numerous than before. With the clustering computed in Table 5, any pair of book is either in the same cluster and part of the same series, or part of different series and in different clusters, with a probability of 83.35%.

These are preliminary results for a proof of concept, and can be improved further.

- We cannot yet suggest a systematic way of pruning the dendrograms to obtain optimal clusters using this method.
- The number of clusters on the K-means analysis was assumed to be the number of distinct authors (10, counting that some books from “The Wheel of Time” were written by a different author). The K-means clustering was tested with other numbers of clusters (from 2 to 15 clusters), and that value did provide an optimum in the separation of the books. This is a weakness of the K-means algorithm, since it assumes that the number of clusters is known in advance

## Conclusion and future work

This paper attempted to replicate the type of network analysis that sociologists perform in their daily studies. Is it important for literature to be respectful of social realities, or are the authors of successful novels imaginative enough in imposing in the same social topology of their history the same fantasy as they create in the fictional worlds in which their characters act?

We proposed a novel algorithm capable of generating multiple types of networks (directed, undirected, weighted, weighted using a sentiment analysis, dynamic) built from novels. Doing so, social networks, alias network, conversation networks now can be generated and analysed. A first social network analysis was also proposed and indicates similarities in the way the social network is presented in novels compared to real life social networks with explanation concerning the differences that might be observed (observation of a power-law degree distribution, high clustering coefficient and an a posteriori preferential attachment verification). Finally, some features were identified that might be relevant in the identification of the author’s style like the dialog spacing and alias network topology.

Moreover, this network analysis provided with a number of characteristics related to the book’s story and the author’s style:

- The dialog spacing frequency (and the resulting threshold value), indicating the relative presence of narration within conversations
- The speaker identification rate, specifically after the metadata extraction step, indicating how much the author reminds their readers of the speakers’ identity
- The degree distribution in the final network
- The average clustering coefficient in the final network, indicating the scope (in time, space and number of protagonists) as well as its relation to the clustering coefficient of a similar random network and its temporal evolution

Once further refined, these features will be combined with other method of stylistic analysis, such as the frequency of function words [64, 71], and the stylistic genome (“stylome”) introduced by van Halteren et al. [72].

As the algorithm is still under development, there are many different ways to improve it and other further analyses are yet to be made considering the vast number of networks that can be generated. A few tasks seem to be most interesting to tackle quickly though. Those concern the formal verification of the character’s identification process as well as the alias table accuracy, solving the coreferences to avoid relying too much on inferences based on the ordering of the dialogs, as well as a sentiment analysis of the dynamic network produced.

With those objectives as a priority, we hope to refine the algorithm further and apply it to other novels as well as other media and try to verify that those novels are built following the same schema of social network.
